# Skin capillary endothelial cells form a network of spatiotemporally conserved Ca^2+^ activity

**DOI:** 10.1101/2025.08.15.669933

**Published:** 2025-08-20

**Authors:** Anush Swaminathan, David G. Gonzalez, Catherine Matte-Martone, Fei Xu, Deandra Simpson, David Monedero-Alonso, Jessica L. Moore, Julia J. Mack, Chen Yuan Kam, Valentina Greco

**Affiliations:** 1 Department of Genetics, Yale School of Medicine, New Haven, CT 06510, USA; 2 Department of Medicine, Division of Cardiology, UCLA, Los Angeles, CA 90095, USA; 3 Molecular Biology Institute, UCLA, Los Angeles, CA 90095, USA; 4 Department of Medicine, Division of Dermatology, UCLA, Los Angeles, CA 90095, USA; 5 Broad Stem Cell Research Center, UCLA, Los Angeles, CA 90095, USA; 6 Departments of Cell Biology and Dermatology, Yale Stem Cell Center, Yale Cancer Center, Yale School of Medicine, New Haven, CT 06510, USA; 7 Howard Hughes Medical Institute (HHMI), Chevy Chase, MD, USA

## Abstract

Ca^2+^ signaling and its regulation are important for endothelial cell (EC) functions, including local blood flow control, mechanotransduction, and barrier function. Yet the spatiotemporal organization of Ca^2+^ activity and its regulation across a vascular plexus is poorly understood in an *in vivo* mammalian context, largely due to technical barriers. To overcome this gap in knowledge, we developed an approach to resolve Ca^2+^ activity with single cell resolution in the skin vasculature of live adult mice by multi-photon imaging. Here, we tracked thousands of Ca^2+^ events in the skin capillary plexus during homeostasis and observed signaling heterogeneity between ECs, with just over half displaying Ca^2+^ activity over minutes. Longitudinal tracking of the same mice revealed that the same ECs maintain Ca^2+^ activity over days to weeks. Interestingly, activity dynamics, such as frequency and event duration, are not conserved at a single cell level but at an EC population level. To identify the molecular underpinning of this spatiotemporal Ca^2+^ activity, we conditionally deleted in ECs the most expressed gap junction protein - Connexin 43 (Cx43). We found that loss of Cx43 initially causes a subset of ECs to display sustained Ca^2+^ activity and biases the dynamics of the whole network towards chronically persistent activity over time. Lastly, through pharmacological targeting of a small panel of known Ca^2+^ mediators, we showed that inhibition of L-type Voltage Gated Ca^2+^ channels largely restores physiological Ca^2+^ activity after loss of Cx43, but has no effect on signaling dynamics in homeostatic settings.

## Introduction

Ca^2+^ is an essential second messenger, mediating tightly regulated signaling pathways to integrate spatial and temporal information across multiple scales, from individual cell transients to multi-cell patterns (1–5). *In vitro*, the temporal dynamics of Ca^2+^ activity within cells differentially encodes downstream functions, enabling its versatility as a signaling pathway (6–11). In excitable tissues *in vivo*, such as basal and lateral amygdala (BLA) after application of a conditioning stimuli, there is considerable overlap in which specific neurons are active over days (12). Moreover, the BLA network displays tightly conserved population-level properties, such as maintenance of total proportion of Ca^2+^ active neurons over time (12). In non-excitable tissues *in vivo*, we demonstrated that cells in the epidermal stem cell compartment engage in both local and large-scale Ca^2+^ activity without any stimuli (4). These tissue-wide analyses revealed that all epidermal stem cells display Ca^2+^ activity within one day during homeostasis. However, *in vivo* Ca^2+^ activity, characteristics, and regulation, across other tissues, especially in mammalian systems, remains relatively unexplored.

Endothelial cells (ECs) are specialized squamous cells that line the lumen of all blood vessels (13). The endothelium regulates key functions in the vasculature through Ca^2+^ activity, such as angiogenesis, vessel tone, local blood flow control, mechanotransduction, and barrier integrity (14–19). *In vivo* work in zebrafish vasculature during development has tracked Ca^2+^ across EC populations and identified spatiotemporally organized Ca^2+^ activity specifically in cells driving vessel remodeling (tip cells) (20). These cells also display variable Ca^2+^ temporal dynamics which shape downstream vessel fates (20). Recently, more studies have focused on mouse brain capillaries -given their role in the exchange of gases, nutrients and metabolic waste- to identify molecular mechanisms driving Ca^2+^ signaling within individual ECs, including IP_3_R-mediated Ca^2+^ release, and how channels including Kir2.1, TRPV4, TRPA1, and Piezo1 shape Ca^2+^ influx (21–25). Yet, there are major gaps in our understanding of Ca^2+^ organization and temporal dynamics during homeostasis across an entire mammalian vascular plexus in the native, unperturbed tissue environment.

Organization of Ca^2+^ activity also involves information exchange between cells, as ECs can coordinate Ca^2+^ activity in response to different homeostatic cues (26–30). While individual ECs can regulate cytoplasmic Ca^2+^ concentrations through a variety of mechanisms, multicellular coordination of Ca^2+^ activity between ECs has largely been studied in the context of gap junction-mediated intercellular communication (31). Gap junctions are intercellular channels that allow for cell-cell communication in all tissues, including the vasculature, through electrical coupling and transfer of second messengers such as IP_3_ (32, 33). Evidence for EC coordination during homeostasis, development and injury indicates that molecular signaling may not only be influenced by direct neighbors but regulated on a tissue-wide scale (34–38). Gap junctions have recently been implicated in coordination of Ca^2+^ signaling networks in other tissues such as epithelial stem cells and blood progenitor stem cells. Loss of Connexin 43 (Cx43) gap junctions leads to tissue-wide uncoupling of coordination across the epidermal stem cell compartment and repeated, more frequent, and longer signaling within restricted neighborhoods (4). In *Drosophila* lymph glands, perturbing pan-gap junctions changes the Ca^2+^ signaling frequency and promotes precocious differentiation of blood progenitors (39).

Despite these important insights, it remains challenging to study Ca^2+^ dynamics in an *in vivo* mammalian system given the technical challenges of tracking ECs and their signaling across space and time. Here, we overcome this challenge by focusing on the skin capillary plexus - due to its critical roles in nutrient and oxygen delivery - and tracking Ca^2+^ dynamics in hundreds of ECs over time through our non-invasive, intravital imaging approach (Figure S1) (4, 36). We observed extensive Ca^2+^ activity across capillary plexus ECs during homeostasis, with patterns of Ca^2+^ conserved spatially within the same cells and temporally at the network level. To probe how the spatiotemporal organization of EC Ca^2+^ activity is coordinated, we conditionally deleted Connexin 43 (Cx43cKO) in ECs, and discovered chronically sustained Ca^2+^ activity and dysregulation of EC temporal coordination across the capillary plexus. Finally, we show that L-type Voltage Gated Ca^2+^ channels (VGCCs) are responsible for aberrant Ca^2+^ entry specifically after Cx43cKO, unlike in the wildtype setting. Altogether, this study defines the spatiotemporal organization and regulation of Ca^2+^ activity by a conserved EC network in the skin capillary plexus.

## Results

### Spatiotemporal analyses of the skin capillary plexus reveal a network of endothelial cells with conserved Ca^2+^ activity over time.

**Figure 1:**
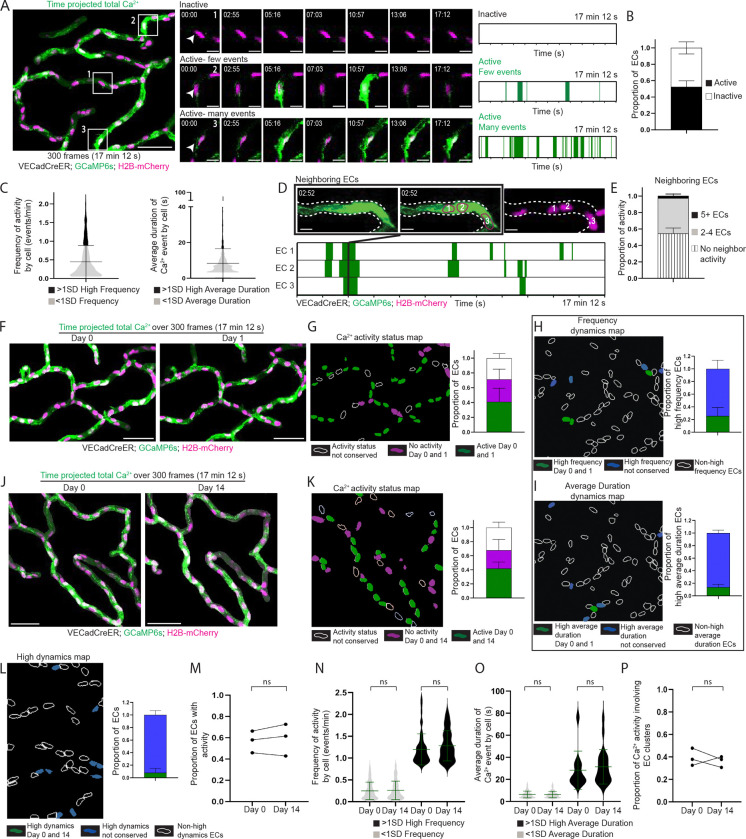
The skin capillary plexus displays Ca^2+^ activity orchestrated by a spatiotemporally conserved network of endothelial cells **(A)** *Left*: Max intensity projection of GCaMP6s signal (green) with H2B-mCherry signal (magenta) from 300 frame (17 min 12 s) recording of skin capillary ECs showing heterogenous Ca^2+^ activity between cells (scale bar: 50 μm). *Mid*: Insets of 3 different regions represent differences in Ca^2+^ activity between cells (scale bar: 10 μm). *Right*: Ca^2+^ events and their durations over the recording time for each of the ECs displayed in the insets (green represents Ca^2+^ activity) **(B)** Proportion of ECs displaying Ca^2+^ activity (active ECs in black and inactive ECs in white). *n=*14 regions from 4 mice. **(C)** Frequency of Ca^2+^ activity per cell (events/min) and average duration (s) of a Ca^2+^ event by cell across 1,765 ECs from 4 mice. EC groups represented by black (>1SD high activity) and grey (<1SD activity) **(D)** *Top*: Ca^2+^ activity occurring simultaneously across 3 ECs (numbered and drawn in magenta outline matching H2B-mCherry). *Bottom*: Ca^2+^ events and their durations over recording time for each EC (scale bar: 10 μm). Black line across plots indicates the timepoint when all 3 ECs simultaneously display Ca^2+^ activity. **(E)** Proportion of Ca^2+^ activity involving various EC cluster sizes. *n* = 14 regions from 4 mice. **(F)** Max intensity projection of GCaMP6s signal with H2B-mCherry signal, with the same regions revisited and recorded 24 hours later (scale bar: 50 μm). **(G)** *Left*: Ca^2+^ activity status map for ECs revisited after 24 hours in (F). Non-conserved activity status (white outline), inactive both days (magenta) and active both days (green). *Right*: Proportion of ECs by conservation of their activity status. Chi-square analysis (observed activity 24 hours after versus random activity as null hypothesis) P<0.0001. *n=*7 regions from 3 mice. **(H)** and **(I)** *Left*: Dynamics map for ECs revisited after 24 hours for conserved high frequency or high average duration behavior on Day 0 and Day 1 (green), not conserved (blue) or not observed (white outline) groups. *Right*: Conservation of >1SD dynamics behavior (green) as a proportion of total high frequency or high average duration ECs. *n=*7 regions from 3 mice. **(J)** Max intensity projection with the same region revisited and recorded 14 days later (scale bar: 50 μm). **(K)** *Left*: Ca^2+^ activity status map for ECs revisited after 14 days in (J). *Right*: Conservation of activity status as a proportion of total ECs. Chi-square (observed activity 14 days after versus random activity as null hypothesis) P<0.0001. *n=*8 regions from 3 mice. **(L)** *Left*: Dynamics map for ECs revisited after 14 days for conserved >1SD high dynamics (including both high frequency and high average duration). *Right*: Conservation of >1SD high dynamics behavior (green) as a proportion of total high frequency and high average duration ECs. *n=*8 regions from 3 mice. **(M)** Proportion of active ECs from 3 mice on Day 0 and Day 14; ns: P>0.05, paired two-tailed Student’s t-test, *n=*457 total cells from 3 mice. **(N)** and **(O)** Frequency of Ca^2+^ activity per cell (events/min) and average duration (s) of Ca^2+^ events on Day 0 and Day 14. black (>1SD high activity) and grey (<1SD activity); ns: P>0.05, unpaired two-tailed Student’s t-test, *n=*265 active cells from 3 mice. **(P)** Proportion of Ca^2+^ activity involving EC clusters from 3 mice on Day 0 and Day 14; ns: P>0.05, paired two-tailed Student’s t-test.

Understanding plexus-wide Ca^2+^ activity is fundamental to reveal how ECs coordinate their signaling to achieve proper vascular function. The superficial nature of the skin capillary plexus makes it especially tractable to longitudinal tracking of an *in vivo* EC population through non-invasive multi-photon microscopy. Here, we aimed to gain a plexus-wide view of Ca^2+^ activity in skin capillaries with single-cell resolution. Towards this goal, we recorded Ca^2+^ activity with simultaneous imaging of Ca^2+^ dynamics and EC nuclei via genetically encoded fluorescent reporters. We used an EC-specific inducible Cre driver under the control of the vascular endothelial cadherin (VECad) promoter to simultaneously recombine a nuclear H2B-mCherry reporter and GCaMP6s, a Ca^2+^ reporter encoding a calmodulin-GFP fusion protein (VECadCreER; GCaMP6s; H2B-mCherry) (Figure S1 A). We focused on a simplified skin model devoid of hair follicles - palmoplantar skin - and recorded Ca^2+^ activity in the capillary plexus (4 to 5 regions per mouse with 75–150 cells in each region) over a period of 17 minutes and 12 seconds (300 frames at 3.44s/frame) in adult (2–4 months old) anesthetized mice via two-photon microscopy. During these timelapse recordings, we observed dynamic and heterogeneous Ca^2+^ activity across the plexus with hundreds of events per region and different levels of Ca^2+^ activity and dynamics between cells (Figure 1 A; Movie 1). To quantitatively analyze the dynamics of the observed Ca^2+^ activity, we devised a computational analysis pipeline that segments individual ECs by using the position of the nuclear H2B-mCherry signal as a proxy for the cell body (Figure S1 A). This enabled us to isolate and analyze changes in GCaMP intensity of each individual cell within the imaging region for the entire duration of the timelapse recording (Figure S1 B e–g). The pipeline determines the number and duration of Ca^2+^ events per cell without being confounded by variable baseline Ca^2+^ levels, through counting any activity above a set threshold as an event (>50% change in average MFI above the minimum MFI) (Figure S1 B; Figure 1 A). We analyzed 1,765 cells across 4 mice and discovered that just over half of ECs (52.4 ± 7.34%) exhibited Ca^2+^ activity during the timeframe of recording (defined as having an ‘active’ cell status, with the others assigned a status of ‘inactive’; Figure 1 B). Using our pipeline, we found that the majority of active ECs display dynamics within a defined range in both frequency (0.445 ± 0.440 events per minute, encompassing 83.9 ± 7.50% of cells) and average duration (8.36 ± 8.15 seconds per event, encompassing 91.3 ± 3.84% of cells) of Ca^2+^ events (Figure 1 C). A small population of ECs displayed activity above that range, showcasing either high frequency (1.31 ± .351 events/minute) or high average duration (29.8 ± 16.4 seconds per event) dynamics (Figure 1 C; Figure S2 A). Lastly, to understand the prevalence of multi-cellular activity, we adapted our pipeline to identify neighboring cells displaying Ca^2+^ activity either within the same time frame or one frame apart, and calculated the number of cells involved (Figure 1 D; Figure S1 A and B a–d). We found that the most represented cluster size for EC Ca^2+^ activity occurred across 2–4 cells (42.8 ± 5.64% of total activity) with a small percentage involving 5 or more cells (2.97 ± 1.32% of total activity) (Figure 1 E).

As we established a quantitative understanding of EC Ca^2+^ dynamics, we next asked how activity status is regulated over longer periods of time. Towards this goal, we leveraged our longitudinal imaging approach that allows us to revisit the same cells over days to weeks. We began with short-term revisits and intriguingly, discovered that not only was the proportion of active and inactive cells largely conserved after 24 hours (58.7 ±14.4% active on Day 0 vs. 52.0 ± 18.8% on Day 1), but activity status was also conserved at the cellular level (71.1 ± 6.03% of cells maintained their activity status after 24 hours) (Figures 1 F and G; Figure S2 B; Movie 2). We also observed that only 28.9 ± 7.89% of high frequency and 17.0 ± 6.30% of high average duration ECs retained their dynamics after 24 hours, indicating that despite maintaining activity status (active vs. inactive), individual ECs largely exhibit different Ca^2+^ dynamics (frequency and average duration) over days (Figure 1 H and I; Figure S2 C).

Next, we asked whether Ca^2+^ activity status is also conserved over a longer time frame. Given our past work demonstrating that ECs remain positionally stable over weeks in adults (36), we revisited the same regions 2 weeks later (Figure S3 A). Strikingly, we observed that a majority (67.8 ± 7.56%) of the same ECs retained their activity status (Figure 1 J–K; Movie 3) even though their dynamic properties were largely not retained (5.58 ± 6.51% retention of high frequency ECs and 0% retention of high average duration ECs) (Figure 1 L). Interestingly, we found that Ca^2+^ dynamics (frequency and average duration), though not conserved at the cellular level, were maintained over weeks at the network level (Figure 1 L–O). Lastly, the proportion of Ca^2+^ activity involving EC clusters was also conserved over weeks (Figure 1 P).

Collectively, our findings demonstrate that Ca^2+^ activity in the skin capillary plexus is dynamic during vascular homeostasis and spatially patterned by an active network of ECs with temporally conserved properties over weeks.

### Loss of endothelial Connexin 43 leads to chronically sustained Ca^2+^ activity impairing long-term network regulation

Our findings that EC Ca^2+^ properties are conserved at the network level over time raise the question of how this is regulated molecularly. Gap junctions are intercellular channels that have established roles in EC communication and functional coupling (40–42). We hypothesized that maintenance of the active network is spatially coordinated by gap junctions. Connexin 43 is a gap junction protein both widely expressed in the vasculature, and the most expressed Connexin isoform in isolated skin ECs (Figure S3 B i) (43, 44). To investigate the role of Cx43 in the regulation of network Ca^2+^ activity, we combined our Ca^2+^ reporter with Cx43 knockout mice (Cx43^fl/fl^) to generate EC conditional knockouts (VECadCreER; GCaMP6s; H2B-mCherry; Cx43^fl/fl^) and compared them to littermate controls (VECadCreER; GCaMP6s; H2B-mCherry; Cx43^+/+^).

**Figure 2:**
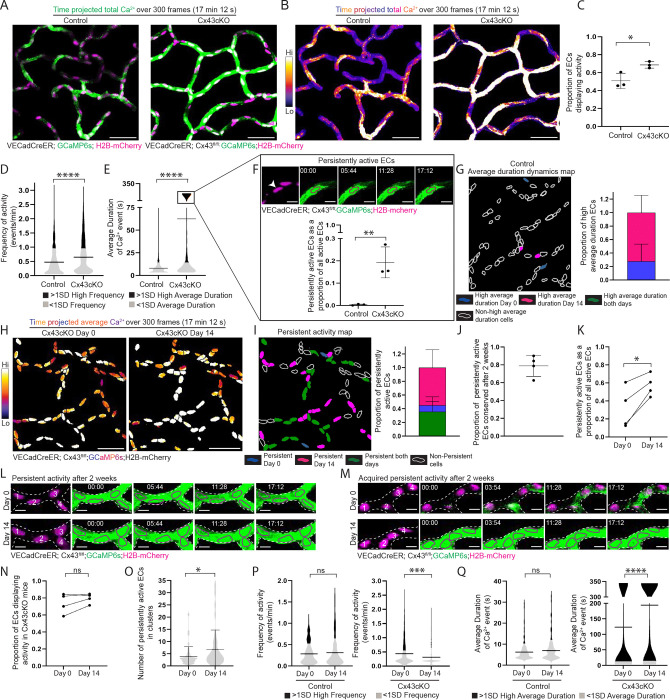
Loss of endothelial Connexin 43 leads to sustained Ca^2+^ dynamics and impairs long-term network regulation **(A)** Max intensity projection of GCaMP6s signal (green) with H2B-mCherry signal (magenta) from 300 frame (17 min 12 s) recording of skin capillary ECs from control (VECadCreER; GCaMP6s; H2B-mCherry) and Cx43cKO (VECadCreER; Cx43^fl/fl^; GCaMP6s; H2B-mCherry) mice (scale bar: 50 μm). **(B)** Max intensity projection of GCaMP6s signal from (A) represented with fire lookup table. Color scale indicates GCaMP6s signal over total duration of recording (scale bar: 50 μm). **(C)** Proportion of active ECs in control and Cx43cKO mice. P=0.0288, unpaired two-tailed Student’s t-test; *n=*10 regions from 3 mice each for control and Cx43cKO. **(D) and (E)** Frequency of Ca^2+^ activity per cell (events/min) and average duration (s) of a Ca^2+^ event by cell respectively for control and Cx43cKO; black (>1SD high activity) and grey (<1SD activity)**.** *n=*570 active ECs from 3 control mice and 837 active ECs from 3 Cx43cKO mice; P<0.0001 for both, unpaired two-tailed Student’s t-test. **(F)** *Top*: Representative EC (drawn in magenta outline corresponding with H2B-mCherry label) displaying persistent activity in Cx43cKO mouse (scale bar: 10 μm). *Bottom*: Persistently active ECs as a proportion of total active ECs in control and Cx43cKO mice; P=0.009, unpaired two-tailed Student’s t-test. **(G)** *Left:* Representative average duration cells map of control ECs revisited after 14 days. High average duration ECs on Day 0 (blue), Day 14 (magenta) or both days (green), and non-high average duration ECs (white outline) *Right:* Bar graph of high average duration ECs on day 0, 14, or both days. *n=*4 regions from 3 mice **(H)** Average intensity projections of GCaMP6s signal over H2B-mCherry region for single-cell resolution (fire lookup table) in Cx43cKO ECs revisited after 14 days (scale bar: 50 μm). Color scale indicates average GCaMP6s signal over total duration of recording. Average intensity projection provides visualization of persistently active ECs in white. **(I)** *Left*: Persistent activity map of ECs revisited after 14 days. Persistently active ECs on Day 0 (blue), Day 14 (magenta) or both days (green), and non-persistently active ECs (white outline). *Right*: Bar graph of persistently active ECs on Day 0, 14, or both days. *n=7* regions from 4 mice. **(J)** Proportion of persistently active ECs conserved after 14 days among revisited mice. **(K)** Persistently active ECs as a proportion of all active ECs, in mice revisited after 14 days; P=0.023, paired two-tailed Student’s t-test. *n=7* regions from 4 mice. **(L)** Representative image of ECs (numbered and drawn in magenta outline of H2B-mCherry signal) maintaining persistent activity after 14 days in Cx43cKO mouse (scale bar: 10 μm). **(M)** Representative image of ECs gaining persistent activity on Day 14 (scale bar: 10 μm). **(N)** Proportion of active ECs from 4 Cx43cKO mice on Day 0 and Day 14; ns: P>0.05, paired two-tailed Student’s t-test, *n=*532 total cells from 4 mice. **(O)** Number of persistently active ECs in clusters, for regions revisited after 14 days; P=0.047, unpaired two-tailed Student’s t-test. **(P)** Frequency of Ca^2+^ activity per cell (events/min) and average duration **(Q)** of Ca^2+^ events by cell (s) on Day 0 and Day 14 of revisited ECs in control and Cx43cKO mice; black (>1SD high activity) and grey (<1SD activity). P<0.0001, unpaired two-tailed Student’s t-test in Cx43cKO mice; *n=*399 active cells from 4 mice**.** ns= P>0.05, unpaired two-tailed Student’s t-test in control mice, *n=*126 active cells from 3 mice.

Cx43cKO and control mice were induced over 4 consecutive days (2mg Tamoxifen daily) and then imaged 3 days later (1 week following the first Tamoxifen injection – referred to as Day 0). Surprisingly, we observed an increase in capillary plexus Ca^2+^ activity in Cx43cKO mice compared to wildtype controls (Figures 2 A–B; Movie 4). This included a significant increase in the proportion of active ECs (68.6 ± 3.71% in Cx43cKO mice vs. 50.8 ± 8.47% in control mice) (Figure 2 C). We also observed that the active network in Cx43cKO mice displayed significantly increased frequency (0.646 ± 0.586 events/min in the Cx43cKO mice vs. 0.473 ± 0.467 events/min in control mice) and a striking ~7-fold increase in the average duration of Ca^2+^ events (62.5 ± 119s in the Cx43cKO mice vs. 8.00 ± 7.33s in control mice) (Figures 2 D–E). Furthermore, we found a group of ECs which displayed significantly long-lasting Ca^2+^ events ranging between 2 and 17 mins, a behavior of persistent activity not detected in control mice (19.3 ± 6.51% of active ECs in Cx43cKO mice vs. 0.324 ± 0.305% of active ECs in control mice) (Figure 2 F).

In contrast to control mice where EC clusters were active simultaneously only for a few frames, Cx43cKO showed “persistently active” EC clusters (61.7 ± 17.3% involved at least 2 cells), simultaneously active for the entire duration of recording (Figure S3 C–E). To understand whether changes in expression of other vascular Connexins (41) may influence Ca^2+^ activity in Cx43cKO mice, we used qPCR to analyze Connexins 37 and 40 expression in isolated skin ECs and found their transcripts to be similarly expressed in Cx43cKO versus control samples, indicating absence of compensatory changes in expression (Figure S3 B ii).

We next asked whether spatiotemporal Ca^2+^ activity and network-level dynamics of ECs is conserved over time in Cx43cKO mice, as was observed under physiological conditions. Revisiting the same cells in the same Cx43cKO mice, we found that the activity status of ECs was spatially conserved on a single cell level after 14 days (78.4 ± 4.84% of ECs retained their status) (Figure S3 F). Furthermore, persistent Ca^2+^ activity was also conserved on a cellular level after 14 days (78.9 ± 11.5% of persistently active ECs on Day 0 retained their behavior on Day 14) (Figures 2 G–J and L; Movie 5). While there was no change in the proportion of active Cx43cKO ECs after 14 days (Figure 2 N), we observed an increase in the proportion of persistently active cells (32.2 ± 22.9% on Day 0 vs. 57.3 ± 12.2% of active ECs on Day 14) (Figures 2 K and M; Movie 5). This was accompanied by an increase in the cluster size of ECs with persistent activity (from 3.85 ± 4 cells on Day 0 to 6.73 ± 7.99 cells on Day 14) (Figure 2 O). While control mice demonstrated conserved dynamics across the network after 14 days, Cx43cKO mice showed a significant increase in average duration (123 ± 156s on Day 0 vs. 194 ± 165 s on Day 14 ) and a significant decrease in frequency (0.445 ± 0.432 events/min on Day 0 vs. 0.319 ± 0.322 events/min on Day 14), consistent with the observed increase in the number of persistently active cells (Figures 2 P and Q).

Our findings show that loss of Cx43 increases plexus-wide Ca^2+^ activity and enriches for a population of persistently active ECs. In addition, we found that loss of Cx43 maintains spatial patterning over weeks, but biases ECs towards longer-lasting Ca^2+^ activity, and accordingly, persistently active ECs become a larger fraction of the vascular plexus over time.

### L-type Ca^2+^ channels sustain elevated Ca^2+^ dynamics following loss of endothelial Connexin 43

Our findings revealed an unexpected increase in Ca^2+^ activity across the EC network after loss of Cx43. To identify what molecular mediators are responsible for this signaling phenotype, we tested a small drug panel of well-known inhibitors of ion channels involved in Ca^2+^ entry. Specifically, we topically applied GSK219 (TRPV4 inhibitor), Dooku1 (Piezo1 inhibitor), Nifedipine (L-type Ca^2+^ channel inhibitor), and DMSO vehicle in Cx43cKO mice and imaged the same capillary regions before and after treatment (within 30–60 minutes) (17, 45, 46). Interestingly, we observed that nifedipine appeared to largely decrease total Ca^2+^ activity and persistent activity in Cx43cKO mice relative to the other compounds (Figure S4).

**Figure 3:**
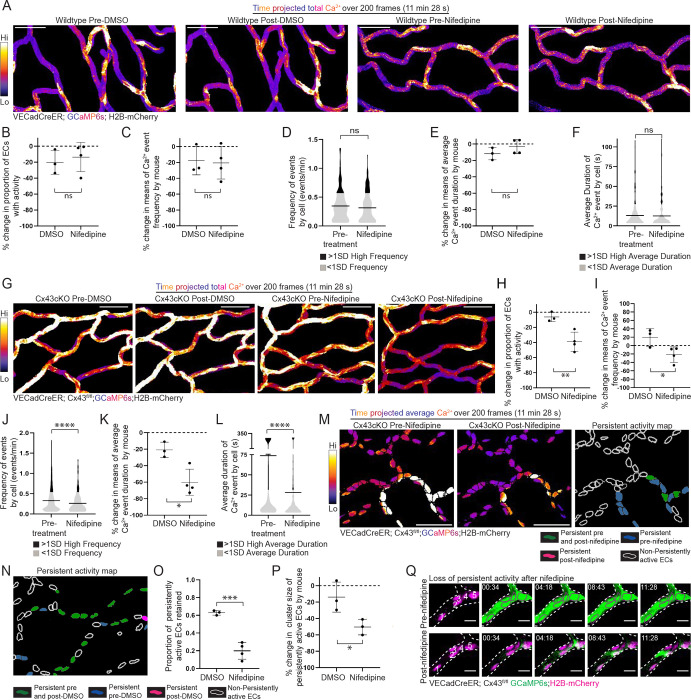
L-type Ca^2+^ channels regulate sustained Ca^2+^ dynamics after Cx43cKO, but do not affect Ca^2+^ activity under physiological conditions **(A)** Max intensity projection of GCaMP6s signal in wildtype mice before and after treatment with either DMSO or nifedipine. GCaMP6s signal is represented with fire lookup table from 200 frame (11 min 28 s) recording of skin capillary ECs before and after treatment. Color scale indicates GCaMP6s signal over recording time (scale bar: 50 μm). **(B)** Percent change in proportion of active ECs in wildtype mice revisited after treatment with either DMSO or nifedipine. ns=P>0.05, unpaired two-tailed Student’s t-test; *n=* 5 regions from 3 mice for DMSO treatment and 6 regions from 4 mice for nifedipine treatment. **(C)** and **(E)** Percent change in frequency (events/min) and average duration (s) dynamics of ECs in wildtype mice revisited after treatment with either DMSO or nifedipine (shown as percentage change of means, refer to Methods under Statistics and Reproducibility). ns=P>0.05, unpaired two-tailed Student’s t-test. **(D)** and **(F)** Frequency of Ca^2+^ activity per cell (events/min) and average duration of Ca^2+^ events by cell (s) of revisited wildtype mice before and after nifedipine treatment. black (>1SD high activity) and grey (<1SD activity); ns=P>0.05, unpaired two-tailed Student’s t-test; *n=*187 active ECs pre-treatment and *n=*173 active ECs after treatment, from 4 mice**. (G)** Max intensity projection of GCaMP6s signal in Cx43cKO mice before and after treatment with either DMSO or nifedipine. (scale bar: 50 μm). **(H)** Percent change in proportion of active ECs in Cx43cKO mice revisited after treatment with either DMSO or nifedipine. P=0.0097, unpaired two-tailed Student’s t-test; *n=*5 regions from 3 mice for DMSO treatment and 6 regions from 4 mice for nifedipine treatment. **(I)** and **(K)** Percent change in frequency (events/min) and average duration (s) dynamics of ECs in Cx43cKO mice revisited after treatment with either DMSO or nifedipine. P=0.04, unpaired two-tailed Student’s t-test, for frequency plot. P=0.013, unpaired two-tailed Student’s t-test, for average duration plot. **(J)** and **(L)** Violin plots showing frequency of Ca^2+^ activity per cell (events/min) and average duration (s) of Ca^2+^ events by cell of revisited regions before and after nifedipine treatment. black (>1SD high activity) and grey (<1SD activity); P<0.0001, unpaired two-tailed Student’s t-test; *n=*358 active ECs pre-treatment and *n=*218 active ECs after treatment, from 4 mice**. (M)** *Left:* Average intensity projections of GCaMP6s signal (fire lookup table) over H2B-mCherry region for single-cell resolution, from regions revisited after nifedipine treatment (scale bar: 50 μm). Color scale indicates average GCaMP6s signal over total recording time. Average intensity projection improves visualization of persistently active ECs in white. *Right*: Map of persistently active ECs after nifedipine treatment. Persistently active ECs pre- and post-nifedipine (green), pre-nifedipine (blue), post-nifedipine (magenta) or non-persistently active ECs (white outline). **(N) and (O)** *Left*: Representative map of persistently active ECs in Cx43cKO mice revisited after treatment with DMSO. *Right*: Proportion of persistently active ECs retained after DMSO versus nifedipine treatments. *n=*5 regions from 3 mice for DMSO and 6 regions from 4 mice for nifedipine treatment. **(P)** Percent change in cluster size of persistently active ECs revisited after treatment with either DMSO or nifedipine; P=0.038, unpaired two-tailed Student’s t-test. **(Q)** Representative images of ECs without persistent activity after nifedipine treatment in a Cx43cKO mouse (scale bar: 10 μm).

Nifedipine is a well-established inhibitor of voltage gated L-type Ca^2+^ channels, blocking Ca^2+^ influx as a response to membrane potential changes (46–49). To first test whether nifedipine would alter Ca^2+^ activity in capillary ECs during homeostasis, we used wildtype (VECadCreER; GCaMP6s; H2B-mCherry) mice and compared the proportion of active ECs, as well as mean values of frequency and average duration by cell per mouse, before and after treatment with nifedipine or DMSO. We did not observe a significant difference in proportion of active ECs (13.7% ± 18.2% decrease after nifedipine treatment vs. 20.2% ± 15.0% decrease after DMSO treatment), frequency (20.6% ± 20.5% decrease after nifedipine treatment vs. 17.8% ± 17.9% decrease after DMSO treatment), or average duration (3.08% ± 8.73% decrease after nifedipine treatment vs. 11.8% ± 7.47 % decrease after DMSO treatment) (Figures 3 A–C and E; Movie 6). In addition, distributions of Ca^2+^ event frequency and average duration by cell were also not significantly different before and after nifedipine treatment, suggesting that L-type channel inhibition does not affect network-wide Ca^2+^ activity in wildtype mice (Figures 3 D and F).

Next, to probe deeper into the effect of nifedipine treatment on Ca^2+^ activity in Cx43cKO mice, we repeated the experiments in Figure S4 utilizing the H2B-mCherry nuclear reporter necessary for single EC segmentation and quantitative analyses. Compared to control DMSO treatment, nifedipine treatment led to decreased proportion of active ECs (39.0% ± 12.6% decrease after nifedipine treatment vs. 6.29% ± 6.23% decrease after DMSO treatment), frequency (21.5% ± 18.5% decrease after nifedipine treatment vs. a 20.0% ± 21.5% increase after DMSO treatment), and average duration (72.8% ± 37.0% decrease after nifedipine treatment vs. 29.3% ± 11% decrease after DMSO treatment) (Figures 3 G–I and K; Movie 7). In addition, the distributions of Ca^2+^ event frequency and duration were decreased after nifedipine treatment compared to before treatment (Figures 3 J and L). We next analyzed the effects of nifedipine on persistently active cells in Cx43cKO mice and confirmed a decrease in persistent activity after treatment (19.9% ± 9.3% of persistently active cells retained after nifedipine treatment vs. 62.9% ± 2.65% after DMSO treatment) and an accompanying decrease in mean cluster size of persistently active ECs (50.5% ± 9.53% decrease after nifedipine treatment vs. 18.4% ± 14.0% decrease after DMSO treatment) (Figures 3 M–Q).

**Figure 4:**
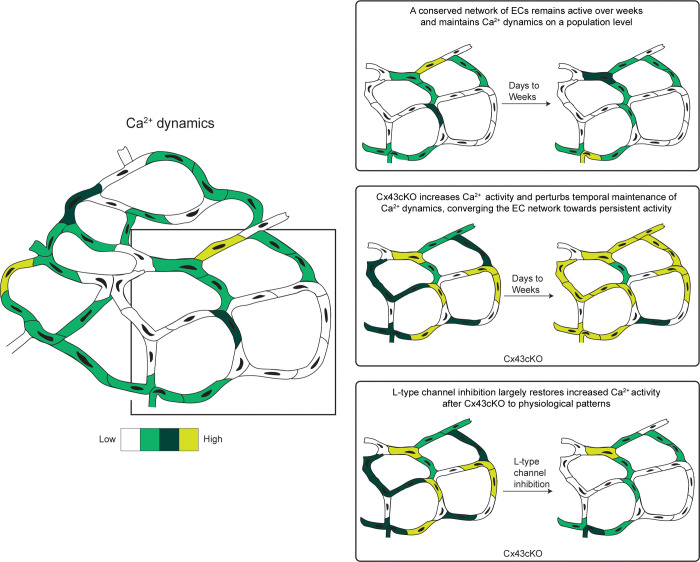
A conserved network orchestrates spatiotemporal Ca^2+^ patterning in capillary ECs over days to weeks and is regulated by Cx43. Model depicting the spatiotemporal characteristics of Ca^2+^ signaling organization in capillary ECs. A spatiotemporally conserved network of ECs orchestrates tissue-wide Ca^2+^ activity over days to weeks and maintains Ca^2+^ dynamics at the population level. Loss of Cx43 increases Ca^2+^ activity and impairs long-term temporal regulation of the EC network, converging on a persistently active phenotype. L-type VGCC inhibition largely restores Ca^2+^ activity in Cx43cKO mice to physiological activity patterns.

These data show that L-type Ca^2+^ channels mediate elevated and persistent EC Ca^2+^ activity after loss of Cx43 (Figure 4). In contrast, these channels do not seem to influence EC Ca^2+^ dynamics in wildtype mice during homeostasis.

## Discussion

How Ca^2+^ activity is organized, maintained, and regulated across a mammalian vascular plexus in its native, unperturbed tissue environment has been poorly understood. To our knowledge, our study represents the first time that Ca^2+^ activity of an *in vivo* population of mammalian ECs has been longitudinally tracked over minutes to days to weeks, demonstrating unexpected spatiotemporal patterns of Ca^2+^ activity and dynamics. Our tissue-wide analysis reveals a network of active cells conserved over time, orchestrating Ca^2+^ activity (Figure 4). Coordinated Ca^2+^ activity as a baseline in adult capillary homeostasis highlights that organized cell networks control Ca^2+^ activity at the tissue-level, and reinforces the notion of the endothelium being a functional syncytium (29, 42, 50). Critically, we discovered that Cx43 functions to constrain Ca^2+^ activity within a dynamic range and maintain network signaling dynamics over the long-term (Figure 4). Our findings elucidate a role of Cx43 as a mediator of cell-cell communication on a larger, tissue-wide scale than previously reported, and as a regulator of temporal coordination of the conserved skin EC network. Furthermore, we found that L-type VGCCs contribute to persistent Ca^2+^ activity after Cx43 loss, uncovering a novel interplay between aberrant L-type VGCC activity and Cx43 dysregulation (Figure 4).

In this study, we discovered and quantified the organization and regulation of homeostatic Ca^2+^ activity in skin vasculature through our unique ability to resolve tissue-wide Ca^2+^ dynamics with single cell resolution *in an intact, uninjured mouse*. The widespread Ca^2+^ activity in skin endothelial cells in physiological conditions (Figure 1) is reminiscent of Ca^2+^ activity observed *in vivo* in other tissues, including mouse skin epidermal stem cells and brain capillaries (4, 17). However, our uncovering of spatiotemporal conservation of Ca^2+^ activity and its large-scale coordination emerged as critical features of skin capillaries (Figure 1). Specifically, the discovery that EC network activity status is maintained on a cellular level and that the distribution of Ca^2+^ dynamics is conserved on a population level is a novel finding across organs and organisms to date. Large-scale coordination between ECs has been shown in periods of extensive vascular remodeling, such as development and injury (34, 36, 37, 51, 52). Our work establishes an unexpected paradigm that Ca^2+^ activity in capillaries is both orchestrated on a tissue-wide scale and is non-random in its spatial patterning. Heterogeneity between ECs in Ca^2+^ activity, which is conserved at a single-cell level over days to weeks, reveals molecular EC heterogeneity between cells in the same vascular compartment. This further builds upon EC heterogeneity that has been extensively described in the same vascular compartment, across the vascular tree, and between organs, to better allow ECs to adapt to their local environments (53–61). Our findings may indicate differences in microenvironment and/or decision-making on a cell-to-cell basis in skin capillaries.

Gap junction Cx43 exerts control over the active network on a temporal scale, regulating how network coordination maintains Ca^2+^ dynamics over time (Figure 2). Ca^2+^ activity in the Cx43ckO capillary plexus converges towards more persistent Ca^2+^ activity without any apparent cell death, while maintaining similar proportions of active ECs. Such perturbed network activity displays remarkable vascular resilience. Given the role of elevated, sustained cytosolic Ca^2+^ in apoptosis and cell death, we anticipate that other molecular mechanisms might function to keep high concentrations of intracellular Ca^2+^ in check (62–64). Previous work has identified the protective effects of acetylcholine, PMCA (Plasma Membrane Ca^2+^-ATPase), and SERCA (Sarco/Endoplasmic Reticulum Ca^2+^ ATPase) upregulation or overexpression in reducing Ca^2+^ overload and promoting cell survival (65–67). Thus, we wonder whether similar changes in our system might buffer Ca^2+^ concentration elevation below the threshold required for cell death. Our findings provide further insight into the spatial range of Cx43 as a mediator of Ca^2+^ communication between cells and indicate that it may serve to coordinate the extent and conservation of Ca^2+^ activity across a network over time.

The phenotype of persistently active cells in Cx43cKO mice (Figure 2) contrasts with *in vitro* and *ex vivo* work in ECs where pharmacological inhibition of pan-gap junctions, or genetic knockout of different Connexin isoforms such as Cx40 leads to decreases in Ca^2+^ activity (26, 29, 68–72). Specifically, in e*x vivo*, both Ca^2+^ activity and spreading between adjacent alveolar capillaries is abolished in EC-specific Cx43 knockout mice, or with a mimetic peptide that blocks Cx43 function (73). While *in vivo* Ca^2+^ activity is yet to be visualized in response to loss of gap junctions, the EC conditional deletion of Cx43, Cx37, and Cx40 leads to phenotypes typically observed downstream of increased Ca^2+^ activity, such as sustained vessel dilation, and increase of plasma nitric oxide (42, 74). Thus, what other features within a native environment with intact plexus architecture may be influencing cytoplasmic Ca^2+^ in ECs after loss of Cx43 remains underexplored.

The novel interplay we uncovered between aberrant L-type VGCC activity and Cx43 dysregulation (Figure 3) provides insight into how voltage gated channels can reshape the EC Ca^2+^ landscape when ECs lack the full complement of gap junction activity. While previous studies have shown conflicting results regarding L-type VGCC expression in capillaries (43, 44, 75–78), the relationship between Cx43 and L-type VGCC activity in capillary ECs requires further investigation. Changes in channel activation independent of expression are possible, since modifications to channel domains on L-type VGCCs are known to modulate the duration of Ca^2+^ influx (79–81). Past studies have also implicated Cx43 gap junctions in maintaining and altering cell membrane potential through their electrical coupling and ion exchange functions (82, 83), potentially allowing for membrane depolarization-mediated activation of L-type VGCCs and Ca^2+^ influx following the loss of Cx43.

Nifedipine may also affect Ca^2+^ activity in Cx43cKO ECs through channel independent or non-cell autonomous mechanisms. Nifedipine can affect VEGF release from ECs, nitric oxide availability, and increase neurotransmitter release in the EC microenvironment, which can all influence EC Ca^2+^ after Cx43cKO (84–87). Furthermore, pericytes are known to influence EC Ca^2+^ activity in other tissues and express L-type VGCCs (88, 89). Indeed, evidence from the brain microvasculature shows that TRPC3 and L-type VGCCs work in concert to affect pericyte function, proposing a model, where ligand-activated TRPC3 provides the membrane depolarization needed for L-type VGCC opening (90). Thus, we acknowledge that future work is needed to fully understand the role of volage gated channels in the vascular capillary plexus.

In conclusion, our work provides insight with unprecedented spatial and temporal resolution into how spatiotemporally conserved networks of capillary ECs regulate tissue-wide Ca^2+^ activity, and provides new concepts to both the vascular biology and Ca^2+^ signaling fields.

### Limitations of the study

In this study, we investigated characteristics and regulation of tissue-level Ca^2+^ activity across capillary ECs *in vivo*, focusing on the skin. One consideration is using a pan-endothelial driver (VECadherin promoter) that does not allow us to discern EC heterogeneity or perturb specific subpopulations of ECs. Drug treatment absorption into the skin can also be variable. This issue was addressed by assessing multiple regions of the plexus within the same mice before and after treatments. Nifedipine used at higher concentrations can also inhibit other membrane ion channels, including T-type VGCCs and Ca^2+^ activated K^+^ channels (KCa_3.1_), though with lower affinity than L-type VGCCs (48, 91, 92).

## Materials and Methods

### Mice

*VECadCreER* (93) mice were obtained from Ralf Adams (Max Planck, Muster, Germany), *Cx43*^*fl/fl*^ (74, 94), *Rosa26-CAG-LSL-GCaMP6s* (95), *Rosa26-CAG-LSL-H2B-mCherry* (96), and *LSL-tdTomato* (95) mice were obtained from The Jackson Laboratory. All experimental mice were bred to a mixed CD1 albino background. Mice from experimental and control groups were randomly selected from either sex for live imaging experiments, and were within an adult age range of 2–4 months. The experiments were not randomized, and all experimentation involving animals were performed under the approval of the Yale School of Medicine Institutional Animal Care and Use Committee (IACUC).

### In vivo imaging

Imaging procedures were adapted from those previously described (97). An isoflurane chamber was used to anesthetize mice, and then the mice were transferred to the imaging stage on a 37 °C heating pad. Mice were maintained on anesthesia throughout the course of recording with vaporized isoflurane delivered by a nose cone (1.25% in oxygen and air). The right paw was mounted on a custom-made stage and a glass coverslip was placed directly against the flat part of the paw. Image stacks were acquired with a A LaVision TriM Scope II (LaVision Biotec) laser scanning microscope with a Chameleon Vision II (Coherent) two-photon laser (using 940 nm for live imaging) and a Chameleon Discovery (Coherent) two-photon laser (using 1120 nm for live imaging). A Nikon 25x/1 water immersion objective was used. Optical sections were scanned with a field of view of 0.3 × 0.3 mm^2^. For all timelapse movies, the live mouse remained anesthetized for the length of the experiment and serial optical sections (3 μm steps for a total stack of 12 μm) were captured at intervals of 3.441s for a total of 200 (11 minutes 28 seconds for topical pharmacological experiments) or 300 frames (17 minutes 12 seconds total recording time). For revisits, the same region of live mouse skin was imaged across intervals of multiple days, and anatomical features of the paw, such as mouse digits, were used as landmarks for finding the same location.

### Image analysis

Raw image stacks were imported into FIJI (ImageJ, National Institutes of Health) for analysis. Max or average (to select for persistently active behaviors specifically) Z stacks of sequential optical sections were chosen. Translational motion correction of timelapse movies was performed on Imaris software.

Analysis of Ca^2+^ for individual ECs: Segmentation of the H2B-mCherry signal was performed via the threshold and masking functions on FIJI. Through a custom MATLAB pipeline, the GCaMP6s fluorescence intensity of each masked region of interest (ROI as a proxy for a cell) every 100 frames was normalized to the minimum fluorescence intensity over that period (Figure S2). Any change in fluorescence over a threshold (50% increase above the minimum fluorescence intensity) was treated as a Ca^2+^ event, and the duration of each event, as well as the number of events per ROI was calculated (Figure S2). Frequency is described as the number of events per minute, with the lowest possible frequency of 0.0588 events/minute (1 event over the 17min 12s recording duration) or 0.001 Hz.

Analysis of Ca^2+^ for EC clusters: Segmentation of the GCaMP6s signal with manual masking was used to segment the vessel surface. Using our custom MATLAB pipeline, we divided the masked vessel surface into individual grids, 0.0076 × 0.0076 mm^2^ and the fluorescence intensity of each grid ROI was normalized every 100 frames by taking the minimum fluorescence value over each period (Figure S2). Any change in GCaMP6s fluorescence over 50% of the minimum fluorescence intensity was identified as a Ca^2+^ event, and the duration of each event, as well as the number of events per ROI was calculated. To analyze EC clusters, adjacent grid ROIs that displayed Ca^2+^ events either at the same frame or one frame apart were considered part of the cluster. The maximum number of grids with connected activity was represented, and the H2B-mCherry segmented ROIs were added to each set of connected grids to determine the number of ECs involved in each cluster (Figure S2).

### Quantitative PCR

RNA from isolated ECs was extracted using Qiagen RNeasy Plus Micro kit (74034). cDNA was made using SuperScript IV First-Strand Synthesis kit (Thermo Fisher 18091050). qPCR utilized FastStart Universal SYBR green Master (Sigma) on the CFX Connect Real-Time PCR Detection System (Bio-Rad). The set of primers used are as follows:

Connexin 37: forward, CCCACATCCGATACTGGGTG; reverse, CGAAGACGACCGTCCTCTG.

Connexin 40: forward, AGGGCTGAGCTTGCTTCTTA; reverse, TTAGTGCCAGTGTCGGGAAT.

Connexin 43: forward, GGTGATGAACAGTCTGCCTTTCG; reverse, GTGAGCCAAGTACAGGAGTGTG.

### Tamoxifen induction

To induce the expression of GCaMP6s, H2B-mCherry, and/or loss of Cx43 expression, *VECadCreER; GCaMP6s; H2B-mCherry* or *VECadCreER; Cx43fl/fl; GCaMP6s; H2B-mCherry* or *VECadCreER; Cx43fl/fl; GCaMP6s* or *VECadCreER; GCaMP6s* or *VeCadCreER; Cx43fl/fl* or *VeCadCreER; tdtomato* or *VeCadCreER* mice were given four doses of tamoxifen (2 mg in corn oil) 4, 5, 6, and 7 days before imaging or tissue collection by intraperitoneal injection (IP).

### Topical drug treatment

For each drug tested, 4–6 capillary regions were imaged for 200 frames (11 min 28 s). To inhibit Piezo1, TRPV4, or L-type Ca^2+^ channels, Dooku1, GSK219, and nifedipine respectively were delivered topically to the paw skin. Nifedipine and GSK219 were dissolved in a 100 mg ml^−1^ stock solution in dimethyl sulfoxide (DMSO) and 30 μl of the mixture was spread evenly on the paw for 15 minutes. Dooku1 was dissolved in a 60 mg ml^−1^ stock solution in dimethyl sulfoxide (DMSO) and 50 μl of the mixture was spread evenly on the paw for 15 minutes. Regions in the paw were then revisited 30–60 minutes after application of the topical agents. A solution of 100% DMSO was used as vehicle control.

### Endothelial cell isolation and sorting

*VECadCreER* or *VECadCreER; tdtomato* mice were induced with tamoxifen 1 week prior to harvesting tissue. Adult paws were collected and placed dermis side down in a 5 mg/ml dispase II solution (MilliporeSigma; 494207800) for 45 minutes at 37 °C. The epidermis was removed, and the dermis was minced finely and transferred to 0.25% collagenase IV (Sigma; C5138) in HBSS (GIBCO;14170–112) solution for at least 90 minutes at 37 °C. The tissue was then passed through an 18-gauge needle (BD; 305195) and washed in FACS Buffer (3% FBS, 2mM EDTA). The suspensions were then centrifuged at 350G for 10 minutes and filtered through a 40 μm filter (Falcon; 352340) before staining with anti-mouse CD31 APC (Biolegend;160210; 1/200) for sorting on a BD FACS AriaII. ECs were sorted based on expression of CD31 (and for *tdtomato* in *VECadCreER; tdtomato mice*).

### Statistics and reproducibility

GraphPad Prism software (GraphPad, Inc.) was used to perform statistical analyses (version 9.2). Parameters are reported in the figure legends, and comparisons between groups were made using paired or unpaired two-tailed Student’s *t* test, or Chi-squared analysis for observed versus expected outcomes (df=1) in revisits. Normality was assumed in data distribution, but not formally tested. Differences between groups were considered significant at P < 0.05 and the data are presented as means ± SD.

High frequency or high average duration EC populations were designated as displaying activity 1 standard deviation (SD) greater than the mean of all active ECs. For wildtype revisits, the value threshold for >1SD dynamics cells was calculated based on frequency and average duration distributions on Day 0. For Cx43cKO mice, the value threshold for >1SD dynamics cells was based on the frequency and average duration distributions of the control mice.

For Chi-squared analysis, expected values of ECs active on Day 0 and then also when revisited (‘random activity’ as the null hypothesis) were assigned based on the percentage of ECs displaying activity on Day 0 (58% for 24-hour revisits, 54% for 14-day revisits, and 74% for Cx43cKO 14-day revisits). These proportions were then compared to the percentage of cells that were observed to maintain their activity status upon revisiting.

To assess changes in frequency and average duration dynamics in revisited regions after pharmacological application of DMSO or nifedipine, the mean values of frequency and average duration were calculated for each mouse before and after treatment, and the percentage change between the pre-treatment and post-treatment values was compared between the two conditions.

## Supplementary Material

Supplement 1

Supplement 2

Supplement 3

Supplement 4

Supplement 5

Supplement 6

Supplement 7

Supplement 8

## Significance Statement

Ca^2+^ signaling in mammalian endothelial cells (ECs) locally regulates blood flow, force sensing, and vessel permeability. In periods of vascular development and repair, there is a need for large-scale coordination in each of those functions. To understand what mechanisms drive collective Ca^2+^ activity during vascular remodeling, we must first address the open question of how tissue-level Ca^2+^ is spatiotemporally organized and regulated during homeostasis. Intravital imaging in skin vasculature of live mice reveals that a conserved set of cells orchestrates tissue-wide Ca^2+^ from minutes to days to weeks. How this network maintains itself over time requires long-range communication through a gap junction protein, Connexin 43 (Cx43). Cx43 dysregulation subsequently recruits L-type Voltage Gated Ca^2+^ channels to reshape the EC Ca^2+^ landscape.
