# Supplementary material for: Skin capillary endothelial cells form a network of spatiotemporally conserved Ca2+ activity
Source: bioRxiv. 2026 Mar 6:2025.08.15.669933. Originally published 2025 Aug 20. Preprint. [Version 2] doi: 10.1101/2025.08.15.669933 (PMC12393453; doi:10.1101/2025.08.15.669933)
Supplement: 1 [file NIHPP2025.08.15.669933V2-supplement-1.pdf]

bioRxiv preprint doi: https://doi.org/10.1101/2025.08.15.669933; this version posted March 6, 2026. The copyright holder for this preprint (which was not certified by peer review) is the author/funder, who has granted bioRxiv a license to display the preprint in perpetuity. It is made available under aCC-BY 4.0 International license.

Figure S1

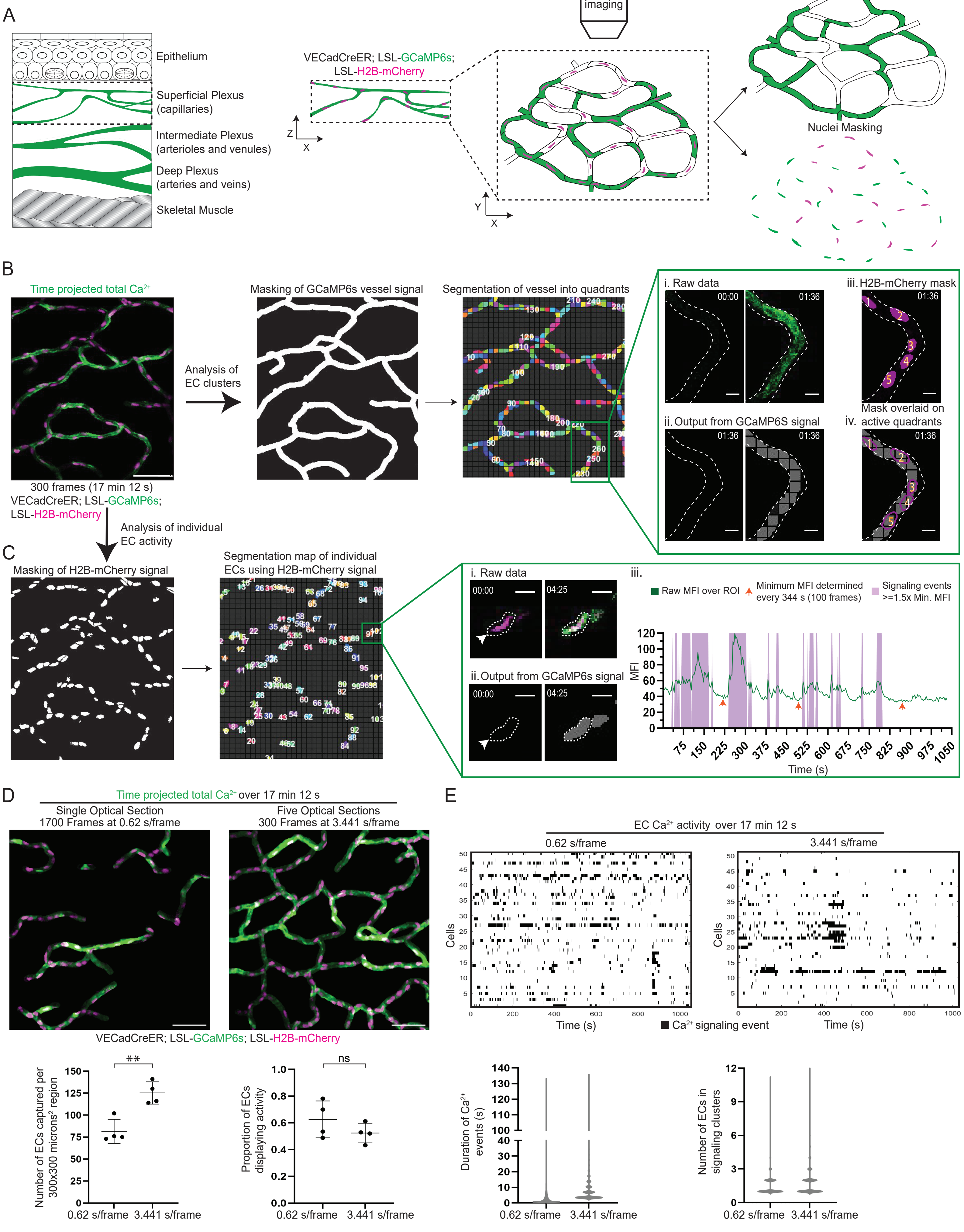

bioRxiv preprint doi: https://doi.org/10.1101/2025.08.15.669933; this version posted March 6, 2026. The copyright holder for this preprint (which was not certified by peer review) is the author/funder, who has granted bioRxiv a license to display the preprint in perpetuity. It is made available under aCC-BY 4.0 International license.

Supplementary Figure Legends

Figure S1

Schematic of intravital imaging and analysis platforms in Ca^{2+} sensor mice and analysis across kinetic scales (A) *Left*: Cartoon representing skin in XZ plane, outlining the superficial capillary plexus. *Middle*: Cartoon of 2-photon imaging over the XY plane of the capillary region, with EC-specific GCaMP6s signal (green) and H2B-mCherry signal (magenta). *Right*: Representation of vessel and nuclei masking from imaged regions. **(B)** *Left:* Max intensity projection of GCaMP6s signal (green) with H2B-mCherry signal (magenta) from 300 frame (17 minutes 12 seconds) recording of skin capillary ECs (scale bar: 50 µm). *Middle:* Masking of vessels based on GCaMP6s signal, and segmentation of vessel mask into 0.0076 x 0.0076 mm^2 quadrants. *Right:* **(i)** Representative images of a Ca^{2+} event, with the **(ii)** pipeline output displaying the number of quadrants involved (scale bar: 10 µm). An event is defined as at least a 50% increase above mean fluorescence intensity (MFI) over the minimum fluorescence for each quadrant. **(iii)** and **(iv)** Adding the H2B-mCherry signal over the pipeline output allows determining the number of ECs involved. **(C)** *Left:* Masking of nuclei based on H2B-mCherry signal as a proxy for individual ECs *Middle:* Segmentation of nuclei mask into individual regions of interest (ROIs) *Right:* **(i)** Representative images of a Ca^{2+} event (scale bar: 10 µm), with the **(ii)** pipeline output displaying an event. An event is defined as at least a 50% increase above MFI over the minimum fluorescence for each ROI. **(iii)** MFI (green) over the ROI over 300 frames of recording (17 minutes 12 seconds), with minimum MFI calculated every 100 frames (orange arrowheads), and overlaid output Ca^{2+} events (purple). **(D)** *Top:* Max intensity projection over 17 min 12 s recording at two frame rates: 0.62 s/frame over a single optical section or 3.44 s/frame over 5 optical sections (scale bar: 50 µm). *Bottom Left:* Number of ECs captured per 0.3 x 0.3 mm^2 region when imaged at 0.62 s/frame and 3.44 s/frame. P=0.003, unpaired t-test; n=11 regions from 4 mice imaged at 0.62 s/frame and 14 regions from 4 mice imaged at 3.44 s/frame. *Bottom Right:* Proportion of active ECs in mice imaged at 0.62 s/frame and 3.44 s/frame. P=0.239, unpaired t-test; n=11 regions from 4 mice imaged at 0.62 s/frame and 14 regions from 4 mice imaged at 3.44 s/frame. **(E)** *Top:* Representative plots of Ca^{2+} signaling events (black) for 50 active ECs imaged at 0.62 seconds/frame and 50 active ECs imaged at 3.44 s/frame. *Bottom:* Duration of Ca^{2+} events and Number of ECs in signaling clusters in mice imaged at 0.62 s/frame and 3.44 s/frame; n=8,733 events from 4 mice imaged at 0.62 s/frame and 6,936 events from 4 mice imaged at 3.44 s/frame.

bioRxiv preprint doi: https://doi.org/10.1101/2025.08.15.669933; this version posted March 6, 2026. The copyright holder for this preprint (which was not certified by peer review) is the author/funder, who has granted bioRxiv a license to display the preprint in perpetuity. It is made available under aCC-BY 4.0 International license.

Figure S2

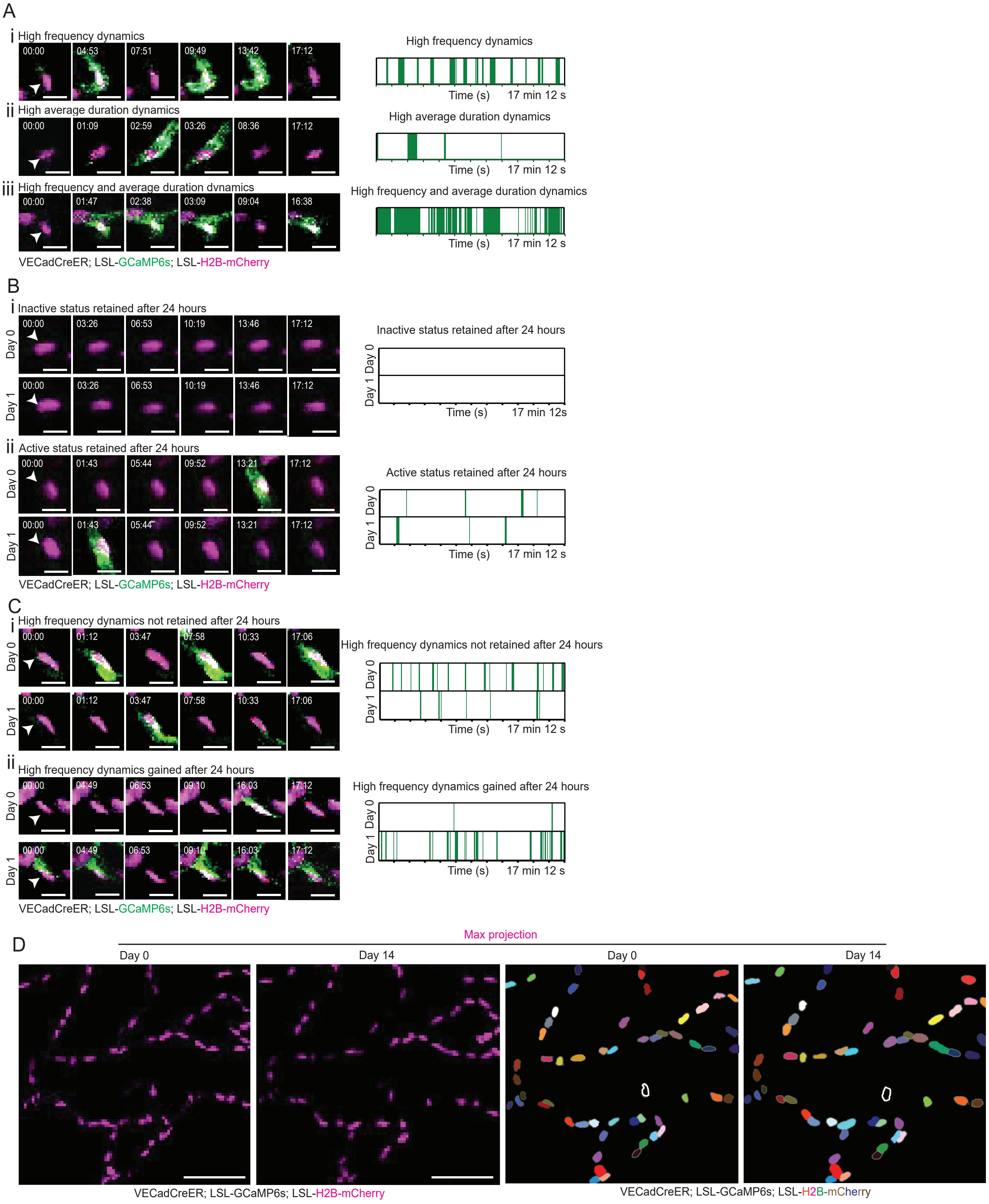

bioRxiv preprint doi: https://doi.org/10.1101/2025.08.15.669933; this version posted March 6, 2026. The copyright holder for this preprint (which was not certified by peer review) is the author/funder, who has granted bioRxiv a license to display the preprint in perpetuity. It is made available under aCC-BY 4.0 International license.

Figure S2

Ca^{2+} signaling dynamics and EC positional stability over time (A) (i) Representative image of EC (nuclei in magenta) displaying high frequency dynamics with plot showcasing Ca^{2+} events (green) and their durations over 300 frames (17 minutes 12 seconds) of recording (scale bar: 10 µm). **(ii)** EC displaying high average duration dynamics. **(iii)** EC displaying high frequency and high average duration dynamics. **(B) (i)** Representative image of EC displaying inactive status on Day 0 and again when revisited 24 hours later, (scale bar: 10 µm). **(ii)** EC displaying activity on Day 0 and again when revisited 24 hours later. **(C) (i)** Representative image of EC losing high frequency dynamics when revisited 24 hours later. (scale bar: 10 µm). **(ii)** EC gaining high frequency dynamics when revisited 24 hours later. (**D)** *Left:* Representative image of region revisited on Day 0 and Day 14 (scale bar: 50 µm). *Right:* ECs multi-color coded, with the same colors on Day 0 and Day 14 corresponding to the same EC.

bioRxiv preprint doi: https://doi.org/10.1101/2025.08.15.669933; this version posted March 6, 2026. The copyright holder for this preprint (which was not certified by peer review) is the author/funder, who has granted bioRxiv a license to display the preprint in perpetuity. It is made available under aCC-BY 4.0 International license.

Figure S3

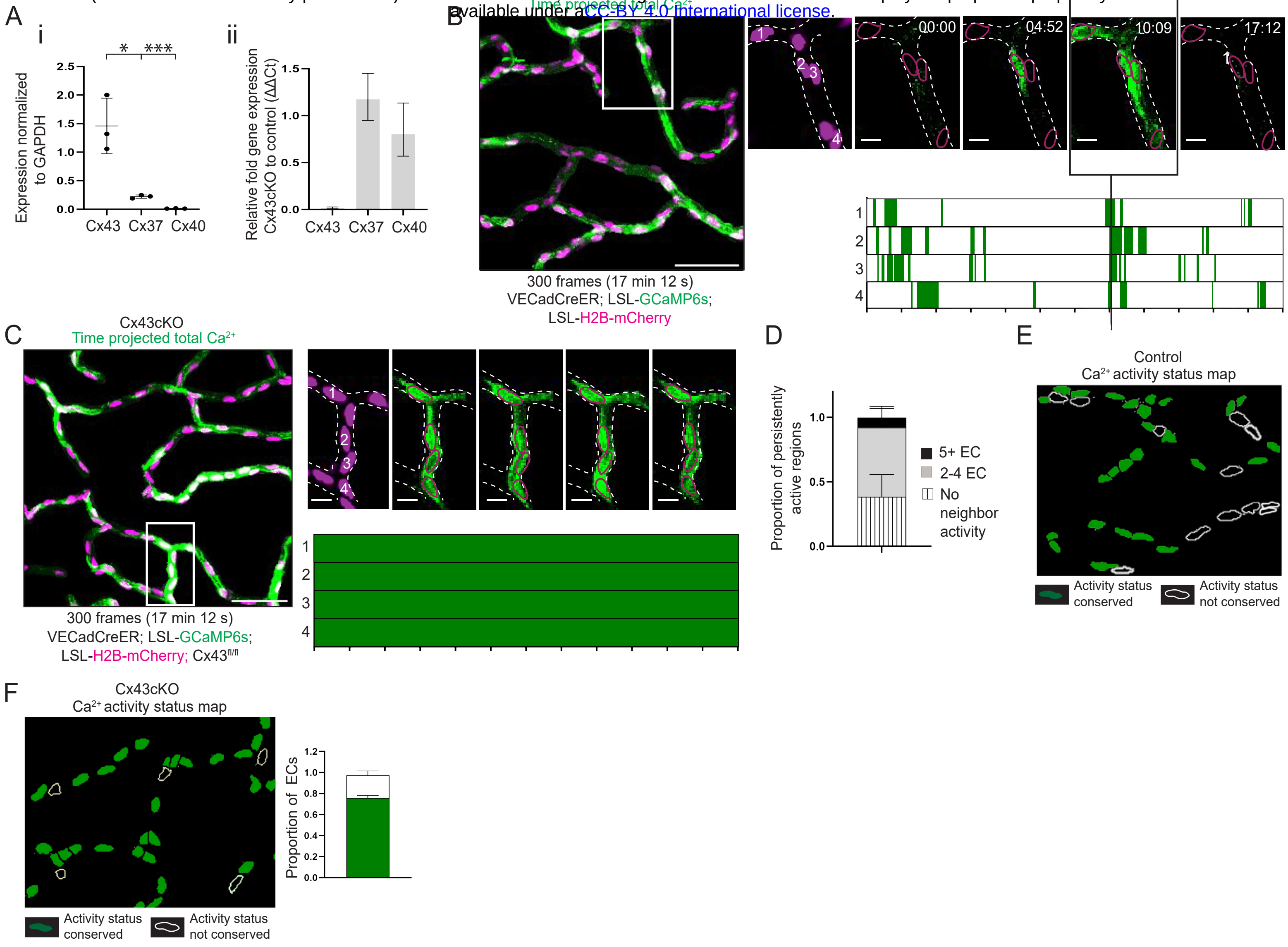

bioRxiv preprint doi: https://doi.org/10.1101/2025.08.15.669933; this version posted March 6, 2026. The copyright holder for this preprint (which was not certified by peer review) is the author/funder, who has granted bioRxiv a license to display the preprint in perpetuity. It is made available under aCC-BY 4.0 International license.

Figure S3

Spatial and molecular analyses of Ca^{2+} dynamics across control and Cx43cKO mice (A) (i) Expression of vascular connexins (Cx43, Cx37, and Cx40) in sorted skin ECs, normalized to GAPDH expression. *n*= 3 mice. **(ii)** Relative expression ($\Delta\Delta C_t$ method) of vascular connexins after Cx43cKO, compared to control mice. Connexin expression in Cx43cKO mice is first normalized to GAPDH, and then to expression in control mice. *n*= 3 Cx43cKO 3 control mice. (**B)** *Left*: Max intensity projection of GCaMP6s signal (green) with H2B-mCherry signal (magenta) from 300 frame (17 minutes 12 seconds) recording of skin capillary ECs in control mice (scale bar: 50 µm). *Right*: Inset of a region with Ca^{2+} activity occurring simultaneously across 4 ECs (numbered and drawn in magenta outline matching H2B-mCherry) (scale bar: 10 µm). *Bottom*: Ca^{2+} events and their durations over recording time for each EC. Black line across plots indicates the timepoint when all 4 ECs simultaneously display Ca^{2+} activity. **(C)** *Left*: GCaMP6s and H2B-mCherry signal from skin capillary ECs in Cx43cKO mice (scale bar: 50 µm). *Right*: Inset of a region with Ca^{2+} activity occurring simultaneously across 4 persistently active ECs (scale bar: 10 µm). *Bottom*: Ca^{2+} events for each persistently active EC. **(D)** Proportion of persistently active regions involving different EC cluster sizes. *n* = 10 regions from 3 mice. **(E)** Ca^{2+} activity status map for control ECs revisited after 14 days. Non-conserved activity status (white outline) and conserved activity status (green). *n*= 4 regions from 3 mice. **(F)** Ca^{2+} activity status map for Cx43cKO ECs revisited after 14 days. Proportion of ECs by their conservation of activity status. Chi-square analysis $P<0.0001$. *n*= 7 regions from 4 mice.

bioRxiv preprint doi: https://doi.org/10.1101/2025.08.15.669933; this version posted March 6, 2026. The copyright holder for this preprint (which was not certified by peer review) is the author/funder, who has granted bioRxiv a license to display the preprint in perpetuity. It is made available under aCC-BY 4.0 International license.

Figure S4

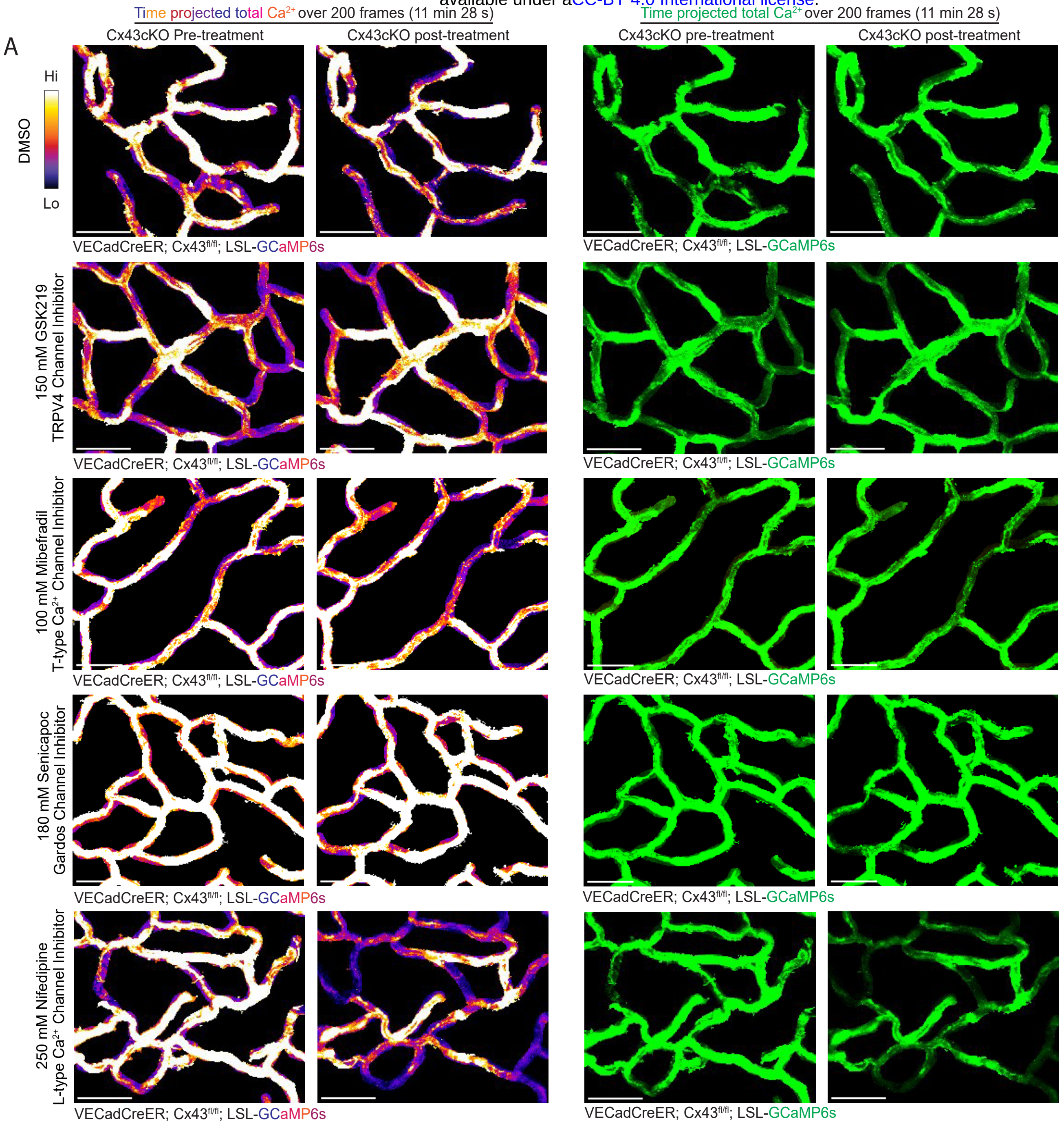

bioRxiv preprint doi: https://doi.org/10.1101/2025.08.15.669933; this version posted March 6, 2026. The copyright holder for this preprint (which was not certified by peer review) is the author/funder, who has granted bioRxiv a license to display the preprint in perpetuity. It is made available under aCC-BY 4.0 International license.

Figure S4

L-type VGCC inhibition, relative to inhibiting T-type VGCCs, KCa3.1 channels, and TRPV4, decreases Ca^{2+} activity after loss of Cx43 (A) Max intensity projection of GCaMP6s signal in green and fire lookup table from 200 frame (11 minutes 28 seconds) recording of skin capillary ECs in Cx43cKO mice before and after treatment with DMSO, GSK219, Mibefradil, Senicapoc, and Nifedipine. Fire lookup table allows for easier visualization of changes in Ca^{2+} signaling intensity. *n*= 3 mice for each condition (scale bar: 50 µm).

Figure S5

bioRxiv preprint doi: https://doi.org/10.1101/2025.08.15.669933; this version posted March 6, 2026. The copyright holder for this preprint (which was not certified by peer review) is the author/funder, who has granted bioRxiv a license to display the preprint in perpetuity. It is made available under aCC-BY 4.0 International license.

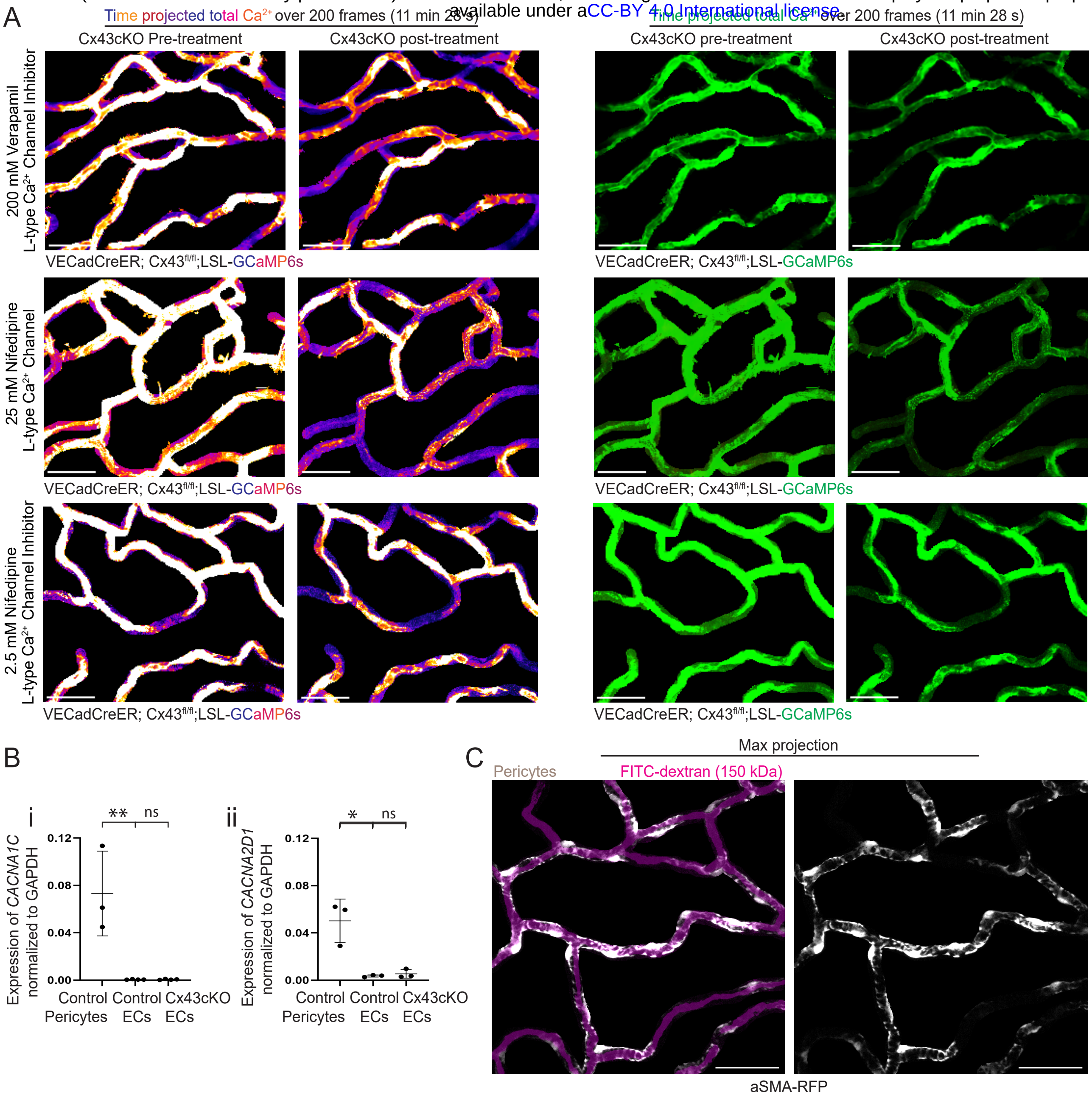

bioRxiv preprint doi: https://doi.org/10.1101/2025.08.15.669933; this version posted March 6, 2026. The copyright holder for this preprint (which was not certified by peer review) is the author/funder, who has granted bioRxiv a license to display the preprint in perpetuity. It is made available under aCC-BY 4.0 International license.

Figure S5

Inhibition of L-type VGCCs specifically decreases Ca^{2+} activity after loss of Cx43 through non-cell autonomous regulation (A) Max intensity projection of recording before and after treatment with verapamil, and nifedipine (10 times and 100 times lower dose). Fire lookup table allows for easier visualization of changes in Ca^{2+} signaling intensity. *n*= 3 mice for each condition (scale bar: 50 µm). **(B) (i)** Expression of *CACNA1C* in sorted skin ECs and smooth muscle actin (SMA+) pericytes during homeostasis, and skin ECs after loss of Cx43, normalized to GAPDH expression. P=0.009 and ns: P>0.05 unpaired t-tests respectively; *n*= 3 aSMA-RFP mice to isolate SMA+ pericytes, n=4 control and Cx43cKO mice. **(ii)** Expression of *CACNA2D1* normalized to GAPDH expression. P=0.0479 and ns: P>0.05 unpaired t-tests respectively; *n*= 3 aSMA-RFP mice, n=4 control and Cx43cKO mice. **(C)** Representative images of capillary region (grey) in aSMA-RFP mice with 150 kDa FITC dextran (magenta) (scale bar: 50 µm).

bioRxiv preprint doi: https://doi.org/10.1101/2025.08.15.669933; this version posted March 6, 2026. The copyright holder for this preprint (which was not certified by peer review) is the author/funder, who has granted bioRxiv a license to display the preprint in perpetuity. It is made available under aCC-BY 4.0 International license.

Figure S6

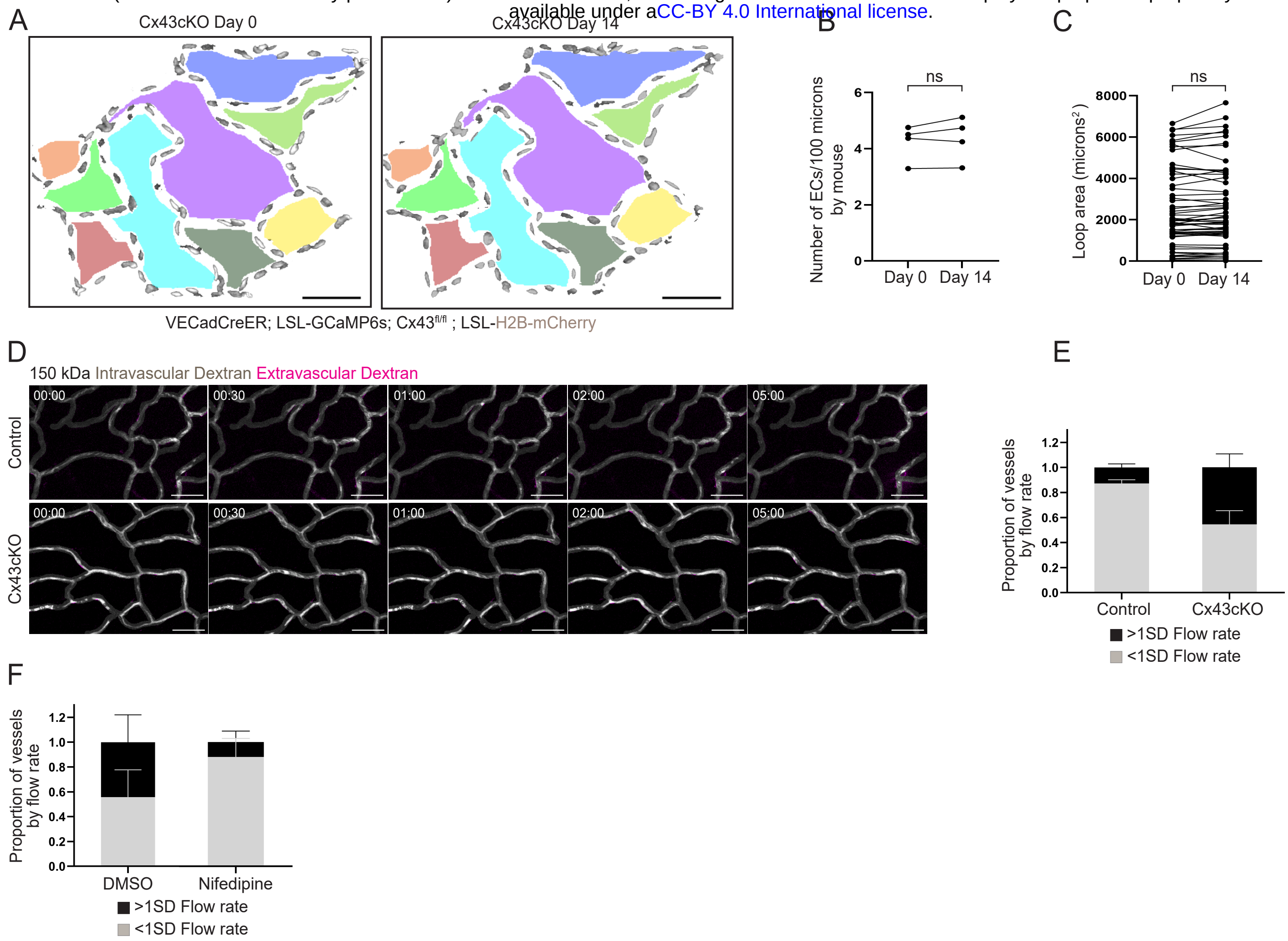

bioRxiv preprint doi: https://doi.org/10.1101/2025.08.15.669933; this version posted March 6, 2026. The copyright holder for this preprint (which was not certified by peer review) is the author/funder, who has granted bioRxiv a license to display the preprint in perpetuity. It is made available under aCC-BY 4.0 International license.

Figure S6

Architectural, barrier, and flow functional analyses downstream of Ca^{2+} elevation after loss of Cx43 (A) *Left:* Max intensity projection of capillary ECs in a Cx43cKO mouse with H2B-mCherry signal (gray) and color-coded architectural loops (scale bar: 50 µm). *Right:* Max intensity projection of the same cells revisited after 14 days, with the same colors corresponding to the same vessel loops (scale bar: 50 µm). **(B)** Number of ECs per 100 µm of the same regions revisited on Day 0 and Day 14 in Cx43cKO mice; ns: P>0.05, paired t-test, *n*=6 regions total from 4 mice. **(C)** Area of the same architectural loops revisited on Day 0 and Day 14 in Cx43cKO mice; ns: P>0.05, paired t-test, *n*=59 total vessel loops from 4 mice. **(D)** Representative single time point images of 150 kDa intravascular dextran (grey) with no extravascular dextran in magenta (scale bar: 50 µm). *n*= 3 mice each for control and Cx43cKO. **(E)** Proportion of vessels by their flow rate in control and Cx43ckO mice, separated by >1SD fast flow vessels (black) and <1SD flow vessels (grey). *n*=45 vessels from 3 mice for each group. **(F)** Proportion of vessels by their flow rate in Cx43cKO mice treated with DMSO or nifedipine. *n*=45 vessels from 3 mice for each group.

bioRxiv preprint doi: https://doi.org/10.1101/2025.08.15.669933; this version posted March 6, 2026. The copyright holder for this preprint (which was not certified by peer review) is the author/funder, who has granted bioRxiv a license to display the preprint in perpetuity. It is made available under aCC-BY 4.0 International license.

Figure S7

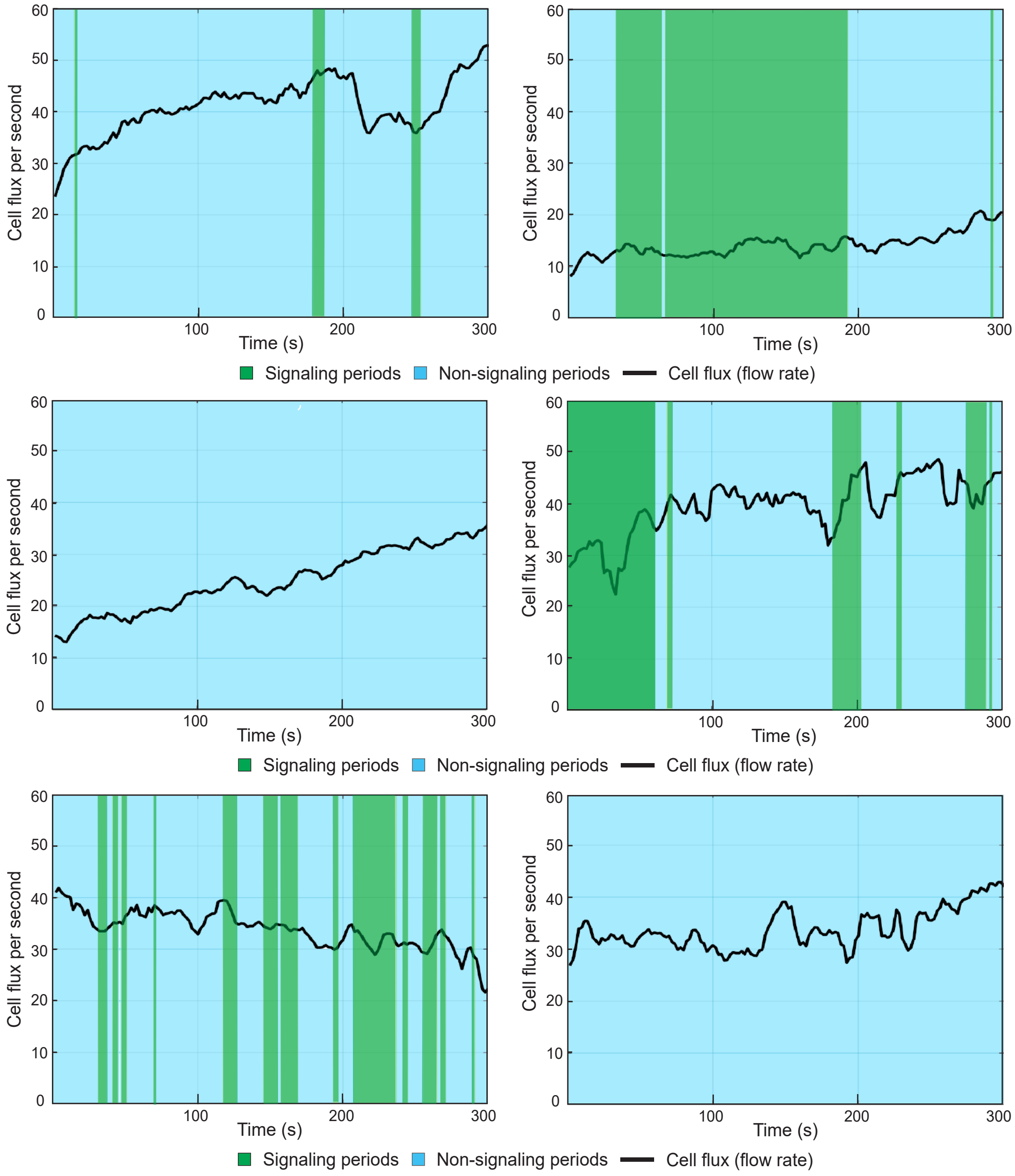

bioRxiv preprint doi: https://doi.org/10.1101/2025.08.15.669933; this version posted March 6, 2026. The copyright holder for this preprint (which was not certified by peer review) is the author/funder, who has granted bioRxiv a license to display the preprint in perpetuity. It is made available under aCC-BY 4.0 International license.

Figure S7

Vessel flow and Ca^{2+} signaling during homeostasis (A) Example graphs across multiple vessels representing cell flux per second or flow rate during line scanning over a 5 min period. Flow rate (black line) is represented over signaling (green) and non-signaling (light blue) time periods.

bioRxiv preprint doi: https://doi.org/10.1101/2025.08.15.669933; this version posted March 6, 2026. The copyright holder for this preprint (which was not certified by peer review) is the author/funder, who has granted bioRxiv a license to display the preprint in perpetuity. It is made available under aCC-BY 4.0 International license.

Movie 1 EC Ca^{2+} activity in skin capillaries is widespread and heterogenous during homeostasis Timelapse of GCaMP6s signal (green) and H2B-mCherry (magenta) from 300 frame (17 minutes 12 seconds) recording of skin capillary ECs, imaged at a frame rate of 3.44 s/frame (scale bar: 20 µm). Timelapse is followed by a max intensity projection of GCaMP6s signal represented with fire lookup table. Color scale indicates GCaMP6s signal over recording time.

Movie 2 EC Ca^{2+} activity status is conserved on a single-cell level after 24 hours *Left*: Timelapse of GCaMP6s signal (green) and H2B-mCherry (magenta) from 300 frame (17 minutes 12 seconds) recording of skin capillary ECs on baseline Day 0, imaged at a frame rate of 3.44 s/frame (scale bar: 20 µm). *Right:* Timelapse of region from Day 0 revisited after 24 hours. Timelapse is followed by a max intensity projection of GCaMP6s signal for Day 0 and Day 1 represented with fire lookup table. Color scale indicates GCaMP6s signal over recording time.

Movie 3 EC Ca^{2+} activity status is conserved on a single-cell level after 14 days *Left*: Timelapse of GCaMP6s signal (green) and H2B-mCherry (magenta) from 300 frame (17 minutes 12 seconds) recording of skin capillary ECs on baseline Day 0, imaged at a frame rate of 3.44 s/frame (scale bar: 20 µm). *Right:* Timelapse of region from Day 0 revisited after 2 weeks. Timelapse is followed by a max intensity projection of GCaMP6s signal for Day 0 and Day 14 represented with fire lookup table. Color scale indicates GCaMP6s signal over recording time.

Movie 4 Cx43cKO leads to sustained EC Ca^{2+} activity *Left*: Timelapse of GCaMP6s signal (green) and H2B-mCherry (magenta) from 300 frame (17 minutes 12 seconds) recording of skin capillary ECs from control mice, imaged at a frame rate of 3.44 s/frame (scale bar: 20 µm). *Right:* Timelapse of capillary ECs from Cx43cKO mice. Timelapse is followed by a max intensity projection of GCaMP6s signal for control and Cx43cKO mice, represented with fire lookup table. Color scale indicates GCaMP6s signal over recording time.

Movie 5 Cx43cKO leads to increase in persistently active ECs after 2 weeks *Left*: Timelapse of GCaMP6s signal (green) and H2B-mCherry (magenta) from 300 frame (17 minutes 12 seconds) recording of skin capillary ECs from Cx43cKO mice on baseline Day 0, imaged at a frame rate of 3.44 s/frame (scale bar: 20 µm). *Right:* Timelapse of region from Day 0 revisited after 2 weeks, on Day 14. Timelapse is followed by an average intensity projection of GCaMP6s signal for Cx43cKO mice on Day 0 and Day 14, represented with fire lookup table. Average intensity projections allow better visualization of persistently active regions in white. Color scale indicates GCaMP6s signal over recording time.

Movie 6 L-type VGCC inhibition does not affect EC Ca^{2+} activity in control mice *Top Left*: Timelapse of GCaMP6s signal (green) and H2B-mCherry (magenta) from 200 frame (11 minutes 28 seconds) recording of skin capillary ECs from control mice prior to DMSO treatment, imaged at a frame rate of 3.44 s/frame (scale bar: 20 µm). *Top Right*: Timelapse of revisited region after DMSO treatment. *Bottom Left*: Timelapse of ECs prior to nifedipine treatment. *Bottom Right*: Timelapse of revisited region after nifedipine treatment. All timelapses are followed by max

bioRxiv preprint doi: https://doi.org/10.1101/2025.08.15.669933; this version posted March 6, 2026. The copyright holder for this preprint (which was not certified by peer review) is the author/funder, who has granted bioRxiv a license to display the preprint in perpetuity. It is made available under aCC-BY 4.0 International license.

intensity projection of GCaMP6s signal represented with fire lookup table. Color scale indicates GCaMP6s signal over recording time.

Movie 7 L-type VGCC inhibition decreases EC Ca^{2+} activity after Cx43cKO *Top Left*: Timelapse of GCaMP6s signal (green) and H2B-mCherry (magenta) from 200 frame (11 minutes 28 seconds) recording of skin capillary ECs from Cx43cKO mice prior to DMSO treatment, imaged at a frame rate of 3.44 s/frame (scale bar: 20 µm). *Top Right*: Timelapse of revisited region after DMSO treatment. *Bottom Left*: Timelapse of ECs prior to nifedipine treatment. *Bottom Right*: Timelapse of revisited region after nifedipine treatment. All timelapses are followed by max intensity projection of GCaMP6s signal represented with fire lookup table. Color scale indicates GCaMP6s signal over recording time.
