# Supplementary material for: Skin capillary endothelial cells form a network of spatiotemporally conserved Ca2+ activity
Source: bioRxiv. 2025 Aug 20:2025.08.15.669933. Preprint. [Version 1] doi: 10.1101/2025.08.15.669933 (PMC12393453; doi:10.1101/2025.08.15.669933)
Supplement: Supplementary file 1 [file NIHPP2025.08.15.669933v1-supplement-8.pdf]

bioRxiv preprint doi: https://doi.org/10.1101/2025.08.15.669933; this version posted August 20, 2025. The copyright holder for this preprint (which was not certified by peer review) is the author/funder, who has granted bioRxiv a license to display the preprint in perpetuity. It is made available under aCC-BY 4.0 International license.

Figure S1

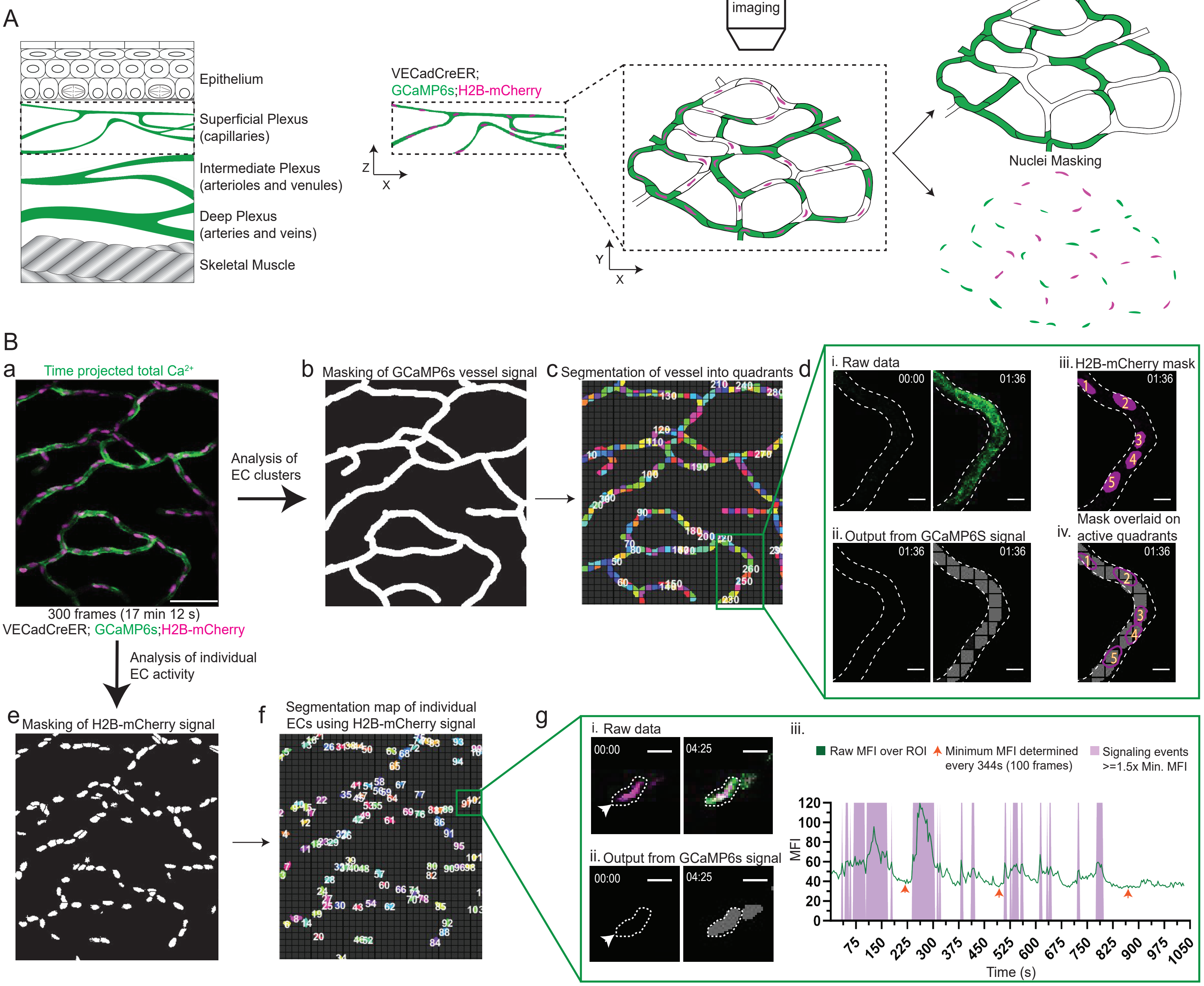

bioRxiv preprint doi: https://doi.org/10.1101/2025.08.15.669933; this version posted August 20, 2025. The copyright holder for this preprint (which was not certified by peer review) is the author/funder, who has granted bioRxiv a license to display the preprint in perpetuity. It is made available under aCC-BY 4.0 International license.

Figure S1

Schematic of intravital imaging and analysis platforms in Ca^{2+} sensor mice (A) *Left*: Cartoon representing skin in XZ plane, outlining the superficial capillary plexus. *Middle*: Cartoon of 2-photon imaging over the XY plane of the capillary region, with EC-specific GCaMP6s signal (green) and H2B-mCherry signal (magenta). *Right*: Representation of vessel and nuclei masking from imaged regions. **(B) (a)** Max intensity projection of GCaMP6s signal (green) with H2B-mCherry signal (magenta) from 300 frame (17 min 12 s) recording of skin capillary ECs (scale bar: 50 µm). **(b) and (c)** Masking of vessels based on GCaMP6s signal, and segmentation of vessel mask into 0.0076 x 0.0076 mm^2 quadrants. **(d) (i)** Representative images of a Ca^{2+} event, with the **(ii)** pipeline output displaying the number of quadrants involved (scale bar: 10 µm). An event is defined as at least a 50% increase above mean fluorescence intensity (MFI) over the minimum fluorescence for each quadrant. **(iii) and (iv)** Adding the H2B-mCherry signal over the pipeline output allows determining the number of ECs involved. **(e)** Masking of nuclei based on H2B-mCherry signal as a proxy for individual ECs **(f)** Segmentation of nuclei mask into individual regions of interest (ROIs) **(g) (i)** Representative images of a Ca^{2+} event (scale bar: 10 µm), with the **(ii)** pipeline output displaying an event. An event is defined as at least a 50% increase above MFI over the minimum fluorescence for each ROI. **(iii)** MFI (green) over the ROI over 300 frames of recording (17 min 12 s), with minimum MFI calculated every 100 frames (orange arrowheads), and overlaid output Ca^{2+} events (purple).

bioRxiv preprint doi: https://doi.org/10.1101/2025.08.15.669933; this version posted August 20, 2025. The copyright holder for this preprint (which was not certified by peer review) is the author/funder, who has granted bioRxiv a license to display the preprint in perpetuity. It is made available under aCC-BY 4.0 International license.

Figure S2

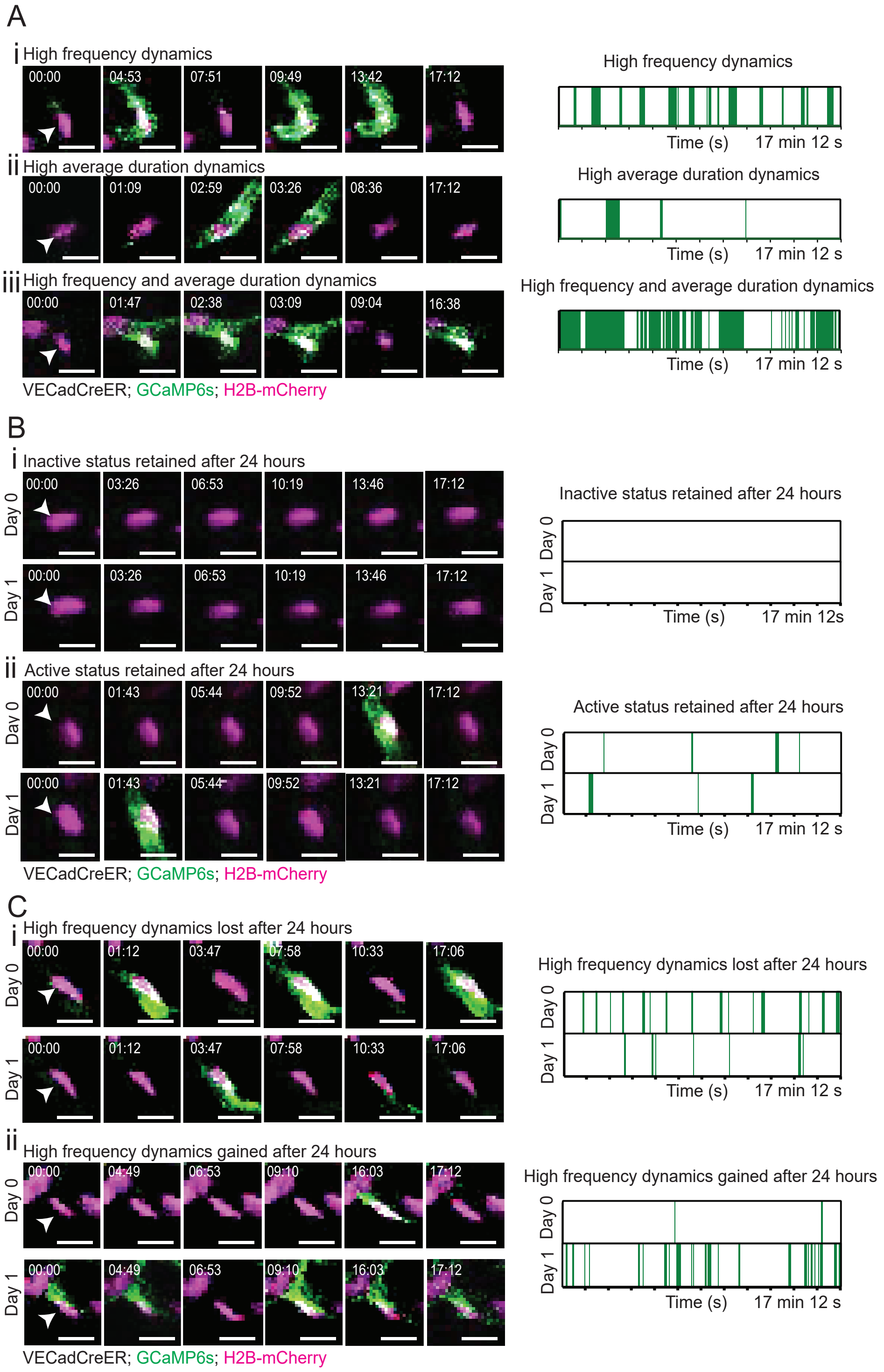

bioRxiv preprint doi: https://doi.org/10.1101/2025.08.15.669933; this version posted August 20, 2025. The copyright holder for this preprint (which was not certified by peer review) is the author/funder, who has granted bioRxiv a license to display the preprint in perpetuity. It is made available under aCC-BY 4.0 International license.

Figure S2

ECs retain activity status but can change dynamics after 24 hours (A) *Top*: Representative image of EC (nuclei in magenta) displaying high frequency dynamics with plot showcasing Ca^{2+} events (green) and their durations over 300 frames (17 min 12 s) of recording (scale bar: 10 µm). *Middle*: EC displaying high average duration dynamics. *Bottom*: EC displaying high frequency and high average duration dynamics. **(B) (i)** Representative image of EC displaying inactive status on Day 0 and again when revisited 24 hours later, (scale bar: 10 µm). **(ii)** EC displaying activity on Day 0 and again when revisited 24 hours later. **(C) (i)** Representative image of EC losing high frequency dynamics when revisited 24 hours later. (scale bar: 10 µm). **(ii)** EC gaining high frequency dynamics when revisited 24 hours later.

bioRxiv preprint doi: https://doi.org/10.1101/2025.08.15.669933; this version posted August 20, 2025. The copyright holder for this preprint (which was not certified by peer review) is the author/funder, who has granted bioRxiv a license to display the preprint in perpetuity. It is made available under aCC-BY 4.0 International license.

Figure S3

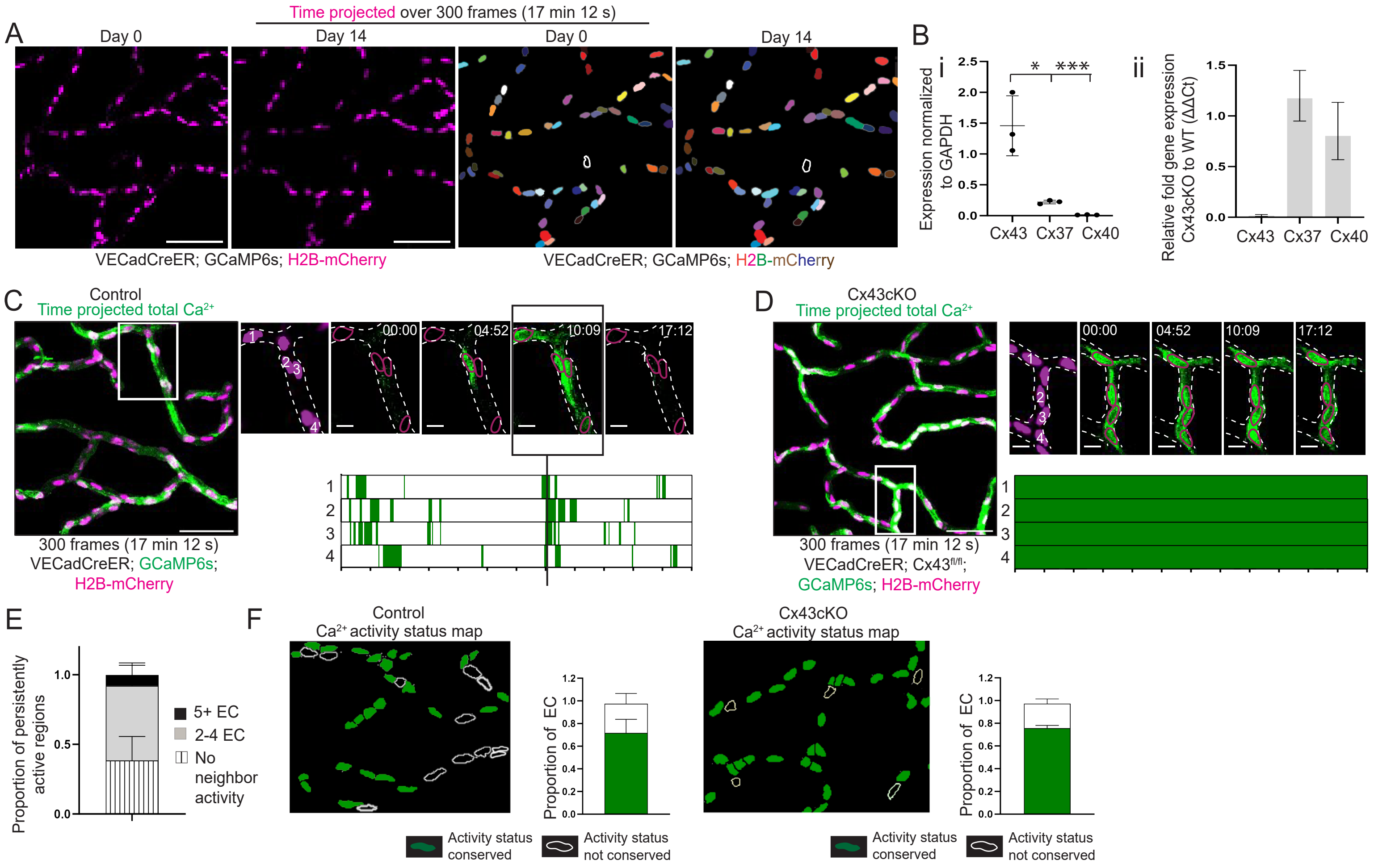

bioRxiv preprint doi: https://doi.org/10.1101/2025.08.15.669933; this version posted August 20, 2025. The copyright holder for this preprint (which was not certified by peer review) is the author/funder, who has granted bioRxiv a license to display the preprint in perpetuity. It is made available under aCC-BY 4.0 International license.

Figure S3

Spatial and molecular analyses of Ca^{2+} dynamics across wildtype and Cx43cKO mice (A) *Left:* Representative image of region revisited on Day 0 and Day 14. *Right:* ECs multi-color coded, with the same colors on Day 0 and Day 14 corresponding to the same EC. **(B) (i)** Expression of vascular Connexins (Cx43, Cx37, and Cx40) in sorted skin ECs, normalized to GAPDH expression. *n*= 3 mice. **(ii)** Relative expression ($\Delta\Delta C_t$ method) of vascular Connexins after Cx43cKO, compared to control mice. Connexin expression in Cx43cKO mice is first normalized to GAPDH, and then to expression in wildtype mice. *n*= 3 Cx43cKO and *n*=3 control mice. (**C**) *Left*: Max intensity projection of GCaMP6s signal (green) with H2B-mCherry signal (magenta) from 300 frame (17 min 12 s) recording of skin capillary ECs in control mice (scale bar: 50 µm). *Right*: Inset of a region with Ca^{2+} activity occurring simultaneously across 4 ECs (numbered and drawn in magenta outline matching H2B-mCherry) (scale bar: 10 µm). *Bottom*: Ca^{2+} events and their durations over recording time for each EC (scale bar: 10 µm). Outline of the EC H2B-mCherry signal is shown in magenta. Black line across plots indicates the timepoint when all 4 ECs simultaneously display Ca^{2+} activity. **(D)** *Left*: GCaMP6s and H2B-mCherry signal from skin capillary ECs in Cx43cKO mice (scale bar: 50 µm). *Right*: Inset of a region with Ca^{2+} activity occurring simultaneously across 4 persistently active ECs (scale bar: 10 µm). *Bottom*: Ca^{2+} events for each persistently active EC. **(E)** Proportion of persistently active ECs involving different cluster sizes. *n* = 10 regions from 3 mice. **(F)** *Left*: Ca^{2+} activity status map for control ECs revisited after 14 days. Non-conserved activity status (white outline) and conserved activity status (green). Proportion of ECs by their conservation of activity status. Chi-square analysis (observed 14 days after versus random activity as null hypothesis) $P<0.0001$. *n*= 4 regions from 3 mice. *Right*: Ca^{2+} activity status map for Cx43cKO ECs revisited after 14 days. Proportion of ECs by their conservation of activity status. Chi-square analysis $P<0.0001$. *n*= 7 regions from 4 mice.

bioRxiv preprint doi: https://doi.org/10.1101/2025.08.15.669933; this version posted August 20, 2025. The copyright holder for this preprint (which was not certified by peer review) is the author/funder, who has granted bioRxiv a license to display the preprint in perpetuity. It is made available under aCC-BY 4.0 International license.

Figure S4

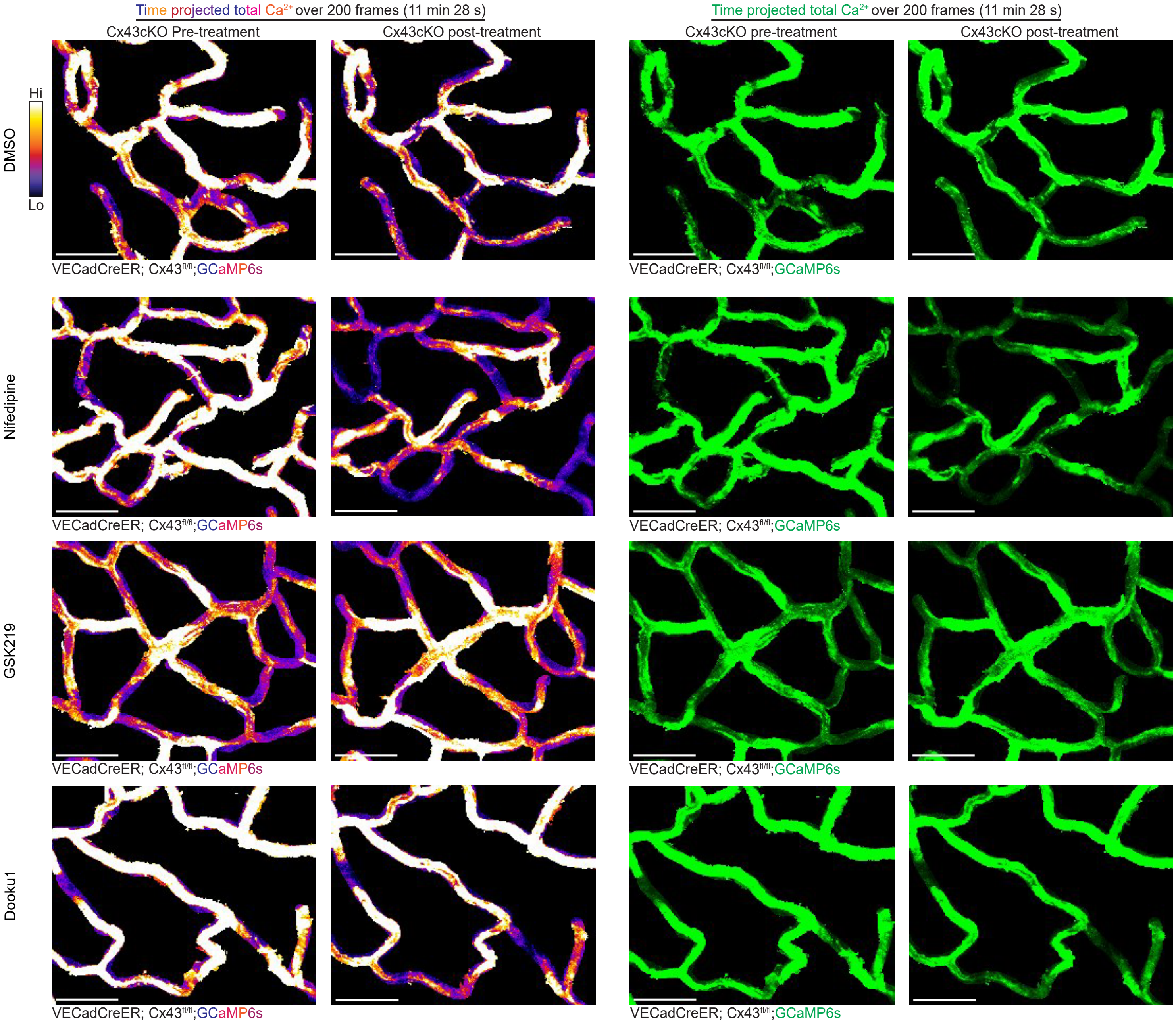

bioRxiv preprint doi: https://doi.org/10.1101/2025.08.15.669933; this version posted August 20, 2025. The copyright holder for this preprint (which was not certified by peer review) is the author/funder, who has granted bioRxiv a license to display the preprint in perpetuity. It is made available under aCC-BY 4.0 International license.

Figure S4

L-type VGCC inhibition, relative to inhibiting Piezo1 and TRPV4, decreases Ca^{2+} activity after loss of Cx43 Max intensity projection of GCaMP6s signal in green and fire lookup table from 200 frame (11 min 28s) recording of skin capillary ECs in Cx43cKO mice before and after treatment with DMSO, nifedipine, GSK219, and Dooku1 (scale bar: 50 μm).

bioRxiv preprint doi: https://doi.org/10.1101/2025.08.15.669933; this version posted August 20, 2025. The copyright holder for this preprint (which was not certified by peer review) is the author/funder, who has granted bioRxiv a license to display the preprint in perpetuity. It is made available under aCC-BY 4.0 International license.

Movie 1 EC Ca^{2+} activity in skin capillaries is widespread and heterogenous during homeostasis Timelapse of GCaMP6s signal (green) and H2B-mCherry (magenta) from 300 frame (17 min 12 s) recording of skin capillary ECs, imaged at a frame rate of 3.441s/frame (scale bar: 20 µm). Timelapse is followed by a max intensity projection of GCaMP6s signal represented with fire lookup table. Color scale indicates GCaMP6s signal over recording time.

Movie 2 EC Ca^{2+} activity status is conserved on a single-cell level after 24 hours *Left*: Timelapse of GCaMP6s signal (green) and H2B-mCherry (magenta) from 300 frame (17 min 12 s) recording of skin capillary ECs on baseline Day 0, imaged at a frame rate of 3.441s/frame (scale bar: 20 µm). *Right:* Timelapse of region from Day 0 revisited after 24 hours. Timelapse is followed by a max intensity projection of GCaMP6s signal for Day 0 and Day 1 represented with fire lookup table. Color scale indicates GCaMP6s signal over recording time.

Movie 3 EC Ca^{2+} activity status is conserved on a single-cell level after 14 days *Left*: Timelapse of GCaMP6s signal (green) and H2B-mCherry (magenta) from 300 frame (17 min 12 s) recording of skin capillary ECs on baseline Day 0, imaged at a frame rate of 3.441s/frame (scale bar: 20 µm). *Right:* Timelapse of region from Day 0 revisited after 2 weeks. Timelapse is followed by a max intensity projection of GCaMP6s signal for Day 0 and Day 14 represented with fire lookup table. Color scale indicates GCaMP6s signal over recording time.

Movie 4 Cx43cKO leads to sustained EC Ca^{2+} activity *Left*: Timelapse of GCaMP6s signal (green) and H2B-mCherry (magenta) from 300 frame (17 min 12 s) recording of skin capillary ECs from control (WT) mice, imaged at a frame rate of 3.441s/frame (scale bar: 20 µm). *Right:* Timelapse of capillary ECs from Cx43cKO mice. Timelapse is followed by a max intensity projection of GCaMP6s signal for control and Cx43cKO mice, represented with fire lookup table. Color scale indicates GCaMP6s signal over recording time.

Movie 5 Cx43cKO leads to increase in persistently active ECs after 2 weeks *Left*: Timelapse of GCaMP6s signal (green) and H2B-mCherry (magenta) from 300 frame (17 min 12 s) recording of skin capillary ECs from Cx43cKO mice on baseline Day 0, imaged at a frame rate of 3.441s/frame (scale bar: 20 µm). *Right:* Timelapse of region from Day 0 revisited after 2 weeks, on Day 14. Timelapse is followed by an average intensity projection of GCaMP6s signal for Cx43cKO mice on Day 0 and Day 14, represented with fire lookup table. Average intensity projections allow better visualization of persistently active regions. Color scale indicates GCaMP6s signal over recording time.

Movie 6 L-type VGCC inhibition does not affect EC Ca^{2+} activity in wildtype mice *Top Left*: Timelapse of GCaMP6s signal (green) and H2B-mCherry (magenta) from 200 frame (11 min 28 s) recording of skin capillary ECs from wildtype (WT) mice prior to DMSO treatment, imaged at a frame rate of 3.441s/frame (scale bar: 20 µm). *Top Right*: Timelapse of revisited region after DMSO treatment. *Bottom Left*: Timelapse of ECs prior to nifedipine treatment. *Bottom Right*:

bioRxiv preprint doi: https://doi.org/10.1101/2025.08.15.669933; this version posted August 20, 2025. The copyright holder for this preprint (which was not certified by peer review) is the author/funder, who has granted bioRxiv a license to display the preprint in perpetuity. It is made available under aCC-BY 4.0 International license.

Timelapse of revisited region after nifedipine treatment. All timelapses are followed by max intensity projection of GCaMP6s signal represented with fire lookup table. Color scale indicates GCaMP6s signal over recording time.

Movie 7 L-type VGCC inhibition decreases EC Ca^{2+} activity after Cx43cKO *Top Left*: Timelapse of GCaMP6s signal (green) and H2B-mCherry (magenta) from 200 frame (11 min 28 s) recording of skin capillary ECs from Cx43cKO mice prior to DMSO treatment, imaged at a frame rate of 3.441s/frame (scale bar: 20 µm). *Top Right*: Timelapse of revisited region after DMSO treatment. *Bottom Left*: Timelapse of ECs prior to nifedipine treatment. *Bottom Right*: Timelapse of revisited region after nifedipine treatment. All timelapses are followed by max intensity projection of GCaMP6s signal represented with fire lookup table. Color scale indicates GCaMP6s signal over recording time.
